# Prognostic value and immune relevancy of a combined autophagy-, apoptosis- and necrosis-related gene signature in glioblastoma

**DOI:** 10.1186/s12885-022-09328-3

**Published:** 2022-03-03

**Authors:** Ying Bi, Zeng-Hong Wu, Fei Cao

**Affiliations:** 1 Department of Neurology, Union Hospital, Tongji Medical College, Huazhong University of Science and Technology, Wuhan, 430022 China; 2 Department of Infectious Diseases, Union Hospital, Tongji Medical College, Huazhong University of Science and Technology, Wuhan, 430022 China

**Keywords:** Glioblastoma (GBM), Cell death index, Immune infiltration, Prognostic, TCGA, CGGA

## Abstract

**Background:**

Glioblastoma (GBM) is considered the most malignant and devastating intracranial tumor without effective treatment. Autophagy, apoptosis, and necrosis, three classically known cell death pathways, can provide novel clinical and immunological insights, which may assist in designing personalized therapeutics. In this study, we developed and validated an effective signature based on autophagy-, apoptosis- and necrosis-related genes for prognostic implications in GBM patients.

**Methods:**

Variations in the expression of genes involved in autophagy, apoptosis and necrosis were explored in 518 GBM patients from The Cancer Genome Atlas (TCGA) database. Univariate Cox analysis, least absolute shrinkage and selection operator (LASSO) analysis, and multivariate Cox analysis were performed to construct a combined prognostic signature. Kaplan–Meier survival, receiver-operating characteristic (ROC) curves and Cox regression analyses based on overall survival (OS) and progression-free survival (PFS) were conducted to estimate the independent prognostic performance of the gene signature. The Chinese Glioma Genome Atlas (CGGA) dataset was used for external validation. Finally, we investigated the differences in the immune microenvironment between different prognostic groups and predicted potential compounds targeting each group.

**Results:**

A 16-gene cell death index (CDI) was established. Patients were clustered into either the high risk or the low risk groups according to the CDI score, and those in the low risk group presented significantly longer OS and PFS than the high CDI group. ROC curves demonstrated outstanding performance of the gene signature in both the training and validation groups. Furthermore, immune cell analysis identified higher infiltration of neutrophils, macrophages, Treg, T helper cells, and aDCs, and lower infiltration of B cells in the high CDI group. Interestingly, this group also showed lower expression levels of immune checkpoint molecules PDCD1 and CD200, and higher expression levels of PDCD1LG2, CD86, CD48 and IDO1.

**Conclusion:**

Our study proposes that the CDI signature can be utilized as a prognostic predictor and may guide patients’ selection for preferential use of immunotherapy in GBM.

**Supplementary Information:**

The online version contains supplementary material available at 10.1186/s12885-022-09328-3.

## Introduction

Glioma is the most common type of primary brain tumors in adults. According to the 2016 World Health Organization Classification of Tumors of the Central Nervous System, the diffuse gliomas include WHO grade II and grade III astrocytic tumors, grade II and III oligodendrogliomas, grade IV glioblastomas, and related diffuse gliomas of childhood [1]. Various grades of gliomas differ considerably in tumor pathology, tumor development, and patient prognosis. Glioblastoma (GBM) is considered the most malignant and invasive primary intracranial tumor, with a high risk of recurrence [2–4]. Patients with GBM have a very poor prognosis, with an average overall survival of merely 12–15 months [5]. In spite of recent advances in standard treatment, including surgery, chemotherapy, radiotherapy, and the achievement in targeted therapies and immunotherapies over the past several years, GBM still carries a dismal prognosis with poor survival [6–10]. Therefore, novel prognostic approaches to pick out patients with high risks are warranted to further help therapeutic options for GBM patients.

Cell death is a critical process that maintains physiological homeostasis in multicellular organisms. Recently, numerous studies have revealed that the tumor microenvironment (TME) could be affected by dying and dead cancer cells for their potent immunomodulatory effects [11, 12]. Dying/death cell leads to redundant bioactive factors release, which can either improve or weaken anticancer immunity. Cell death can also result from severe conditions existing in the TME and may significantly alter tumor progression. Researches established that multiple cell death pathways tended to play a part in the treatment response of tumors [13]. The three classically known cell death pathways are autophagy, apoptosis, and necrosis [14, 15]. Autophagy, the process of self-degradation of cellular components, is upregulated when stimulated by extra- or intracellular stress and signals, such as starvation and growth factor deprivation [16]. Consequently, the chronic stress induction can cause irreversible damage, leading to apoptosis or necrosis [17]. Apoptosis is a programmed cell death process with distinct morphological characteristics and energy-dependent biochemical mechanisms [18]. It represents a critical pathway for eliminating cells that are not vital and protects against cells that have received significant genotoxic damage, and is instrumental in immune function [19]. Necrosis, the aftereffect of irreversible cellular damage, is recognized by the rapid destruction of plasma membrane followed by cytoplasmic leakage and the spilling of inflammatory cellular contents into the TME [20]. In short, the cell death processes dysregulation can significantly affect tumorigenesis.

GBM is a highly heterogeneous tumors with multiple subtypes, functionally different for their specific immunological landscapes, such as differences in T cell infiltration and macrophage composition, which require different treatment regimens [21, 22]. Immune checkpoints, widely studied in recent years, are immunosuppressive molecules that avoid normal tissue damage and destruction primarily by modulating the immune response of T cells. Therefore, activating immune checkpoints may cause immune tolerance during tumor progression. Immune checkpoint inhibitors (ICI) can evade anti-tumor immune response, act on the tumor, and restrict its growth. The most effective ICI, anti-PD-1/PD-L1, has been approved in non-small cell lung cancer, colon cancer, and melanoma [23]. However, recent clinical trials indicated that anti-PD-1/PD-L1 treatment might not benefit the clinical outcome of GBM without patients’ selection [24]. Besides, contrary to other cancers, there is still no immunotherapy approved by Food and Drug Administration (FDA) for GBM. One of the arguments challenging GBM immunotherapy is its highly immunosuppressive TME. Thus, identifying regulators of the brain TME could help discover promising new targets for therapeutic intervention. Studies analyzed current clinical trial failures and demonstrated that biomarkers for appropriate patient selection for immunotherapy appeared hopeful in GBM treatment [25, 26]. Recently, several novel prognostic markers for GBM patients have been identified through multiomic analysis and differential expression profiles. However, most of these studies are mathematical analyses based on whole-scale genetic or transcriptomic data, and there is still a lack of specific research on multiple biological pathways [27–29]. Therefore, comprehensive recognition of the characteristics of TME cell infiltration mediated by multiple cell death pathways is needed to deepen our understanding of TME immune regulation and help design enhanced treatment for GBM patients.

In this study, GBM patients were stratified based on a combination of autophagy-, apoptosis- and necrosis-related gene signatures along with the characteristics of their immune response to facilitate the prediction of individualized survival and a superior treatment scheme.

## Materials and methods

### Patient population and multiomic data acquisition

The genomic expression and clinical data of GBM patients in the TCGA database were retrieved from GlioVis (http://gliovis.bioin fo.cnio.es/) [30]. The RNA sequencing data of the Illumina HiSeq 2000 platform and the clinical data were accessed from the Chinese Glioma Genome Atlas (CGGA) database (http://www.cgga.org.cn) [31]. We included 518 GBM patients from TCGA and 137 patients from the CGGA database after excluding those without survival information. The data in the TCGA database were analyzed as the training cohort, and data from the CGGA dataset were used for validation. The Trans Per Million values of RNA-Seq and robust multichip analysis-processed values of microarray data were log2 transformed and then normalized by the scale method in R to make the data comparable between platforms [32]. Furthermore, 505 GBM patients’ copy number alteration (CNA) data were obtained from the TCGA database. Using the RCircos package in R, Circos plots visualized the CNA summary results and determined chromosomal alterations [33]. Additionally, the somatic mutation data of 390 GBM patients were acquired on the basis of the whole-exome sequencing platform from the TCGA database. The data were analyzed and uncovered by utilizing the maftools package in R [34].

### Gene Expression Analysis to Determine Cell Death Index (CDI)

To clarify the prognostic association of cell death-related genes in GBM, autophagy-, apoptosis- and necrosis-related gene lists were accessed from the Gene Ontology (GO) and Kyoto Encyclopedia of Genes and Genomes (KEGG) databases through the Gene Set Enrichment Analysis (GSEA) website(https://www.gsea-msigdb.org) [35]. The KEGG dataset of apoptosis-related genes (*n* = 87) (Table S1) and GO gene list of necrosis (*n* = 49) (Table S2) were obtained. Autophagy-related genes were downloaded from the GO dataset and the Human Autophagy Database (HADb, http://www.autop hagy.lu/index.html). The two gene sets were merged into one(*n* = 495) (Table S3). Univariate Cox regression models were used in each cell-death pathway to screen genes associated with OS in the TCGA datasets. The prognostic gene combination for establishing the index was screened out with LASSO regression. To further determine the optimal genes, a multivariate cox regression model was then performed using the “step” function in R. Subsequently, 140 patients, with the highest or lowest expression level of specific pathway genes, were selected from each cell death group. Significant gene signatures from individual cell death pathways were chosen to create a combined prognostic model to construct a cell death index (CDI). The latter was formed on the basis of a linear combination of the regression coefficient acquired from the multivariate Cox regression model and the genes expression levels. The CDI formula was calculated as follows: Risk score = (exprgene1 × Coefgene1) + (exprgene2 × Coefgene2) + … + (exprgene n × Coefgene n). GBM patients were assigned to the low risk and high risk groups according to the median value of the risk scores. Kaplan–Meier survival analyses were conducted to compare the overall survival (OS) and progression-free survival (PFS) in the two groups. The Kaplan–Meier (K-M) method and ROC were performed to evaluate the index efficiency. From 518 patients, 40 patients demonstrated the highest expression level of cell death-related genes, and 40 patients with the lowest expression level of cell death-related genes were picked for further analysis.

### Differential analysis of the high and low CDI groups

The differentially expressed genes (DEGs) between the high and low CDI groups were determined using the limma package in R with conditions of adjusted *P* < 0.05 and |fold change (FC)|> 1 [36]. The volcano plot was constructed by using the ggplot2 package in R.

### Clinico-Pathological Analysis and Cox-Proportional Hazard

Pearson’s chi-square (χ2) test was conducted to compare categorical variables of clinico-pathological characteristics between groups. Univariable and multivariable Cox proportional hazards models were carried out to assess the performance of the CDI in predicting prognosis. The hazard ratios (HR) with 95% confidence intervals (CI) were based on OS.

### Evaluation of Cytokines

To assess the immune activity of GBM patients, cytokine gene list was acquired using the keyword: ‘KEGG cytokine-cytokine receptor interaction’ (*n* = 265 genes) (https://www.gsea-msigdb.org) [37]. The differential expression of cytokines between high and low risk groups of individual cell death pathway and functional enrichment analysis was conducted in the web-based application of STRING ver.11.0 (http://stringdb.org) [38].

### Estimation of TME (Tumor Immune Environment) cell infiltration

Immune infiltration information, including macrophages, neutrophils, B cells, CD4 + T-cells, CD8 + T-cells, and dendritic cells, etc., were accessed based on the tumor immune estimation resource (TIMER2.0) (http://timer.cistrome.org/) [39]. Single-sample geneset enrichment analysis (ssGSEA) was utilized to analyze the subgroups of tumor-infiltrating immune cells between the high CDI and low CDI groups of individual cell death pathway and explore their immune function [40]. At the same time, CIBERSORT [41], xCell [42], MCP-counter [43], quanTIseq [44] and TIMER [45] algorithms were compared between the two groups, and a heatmap was used to display their differences in the immune response. The correlation between tumor immune cell infiltration and the CDI was analyzed to investigate the performance of CDI in the TME of GBM.

### Assessment of the Role of CDI in Immune Checkpoint Blockade (ICB) treatment

Recent researches reported that the expression level of ICB key targets might have a close association with the clinical outcome of ICI [46]. Therefore, the potential immune checkpoints were derived from previous studies [47]. To evaluate the role of CDI in ICB therapy of GBM, we performed correlation analysis between the gene signature and expression level of these ICB key targets.

### Functional enrichment analysis

Functional enrichment on gene level was completed by using the g:Profiler program (https://biit.cs.ut.ee/gprofiler) [48]. It interprets and maps genes to the corresponding enriched pathways based on well-established data sources. The search tool for the Retrieval of Interacting Genes/Proteins (STRING) database was utilized to conduct the protein–protein interaction (PPI) network to uncover the relationships of DEGs [49]. Cytoscape (Ver 3.8.2) and the plugin of Cyto-Hubba were used for visualizing the PPI network and identifying the top 100 highly connected protein nodes (hubs) by degree, betweenness centrality, and closeness centrality of DEGs [50].

### Gene set variation analysis (GSVA)

GSVA was carried out, using the “GSVA” package, to assess the difference in the biological process of the CDI risk groups [51]. Differential analysis of the enrichment scores of KEGG pathways between the high and low risk groups was performed using the limma package in R [35, 36, 52, 53]. The gene sets of “c2.cp.kegg.v7.4.symbols” were retrieved from the MSigDB database for analysis. The molecular pathways enriched differentially between the two groups were determined by |log2FC|> 0.1 and adjusted *P* < 0.05.

### Connectivity Map (CMap) analysis

We used the CMap database (https://clue.io/) to investigate candidate compounds targeting the molecular pathways and genes associated with CDI for GBM patients [54]. The Connectivity Map (CMap) analysis can also display the mechanism of action (MoA) of compounds. The top 148 most upregulated genes (*P* < 0.01) and top 148 most downregulated genes (*P* < 0.01) between the high and low CDI groups were utilized to inquire the CMap database. Potential compounds were identified by the most significant highly expressed genes of each group. The compounds enrichment scores were obtained, and candidate therapeutic drugs for the high risk group were screened out by a negative enrichment score.

## Statistical analysis

All the statistical analyses of this study were executed by the R 4.0.4 software, GraphPad Prism (version 7 GraphPad Software), and SPSS 23.0. To compare the clinico-pathological parameters between groups, the independent Student’s t test was utilized for continuous data, while the Pearson’s chi-square (χ2) test was utilized for categorical data. Statistical differences were compared by the Wilcoxon and Kruskal–Wallis H tests. A two-tailed p-value < 0.05 was considered statistically significant.

## Results

### Construction of the cell death index

**Fig. 1:**
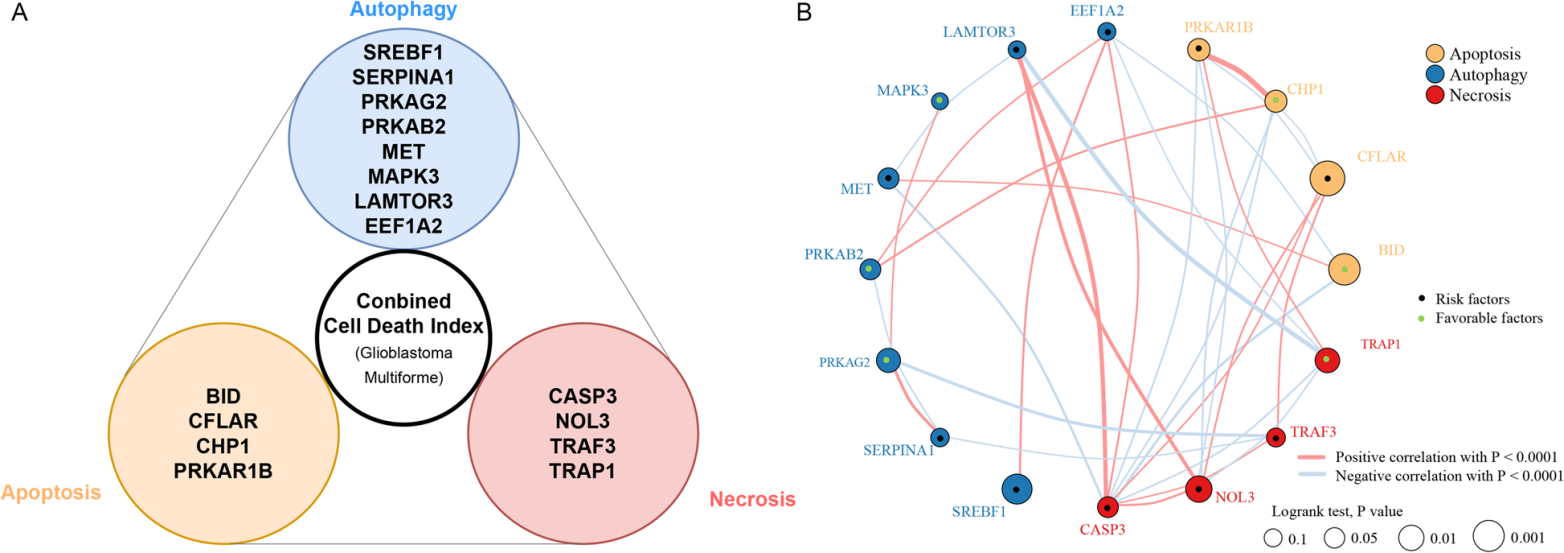
**A**) Combined cell death index (CDI) was generated, which included the highest expression of genes involved in autophagy, apoptosis, and necrosis. **B**) The interaction between CDI signature genes in GBM. The circle size represented the effect of each signature gene on the prognosis, and the range of values calculated by Log-rank test was *p* < 0.001, *p* < 0.01, *p* < 0.05 and *P* < 0.1, respectively. The Autophagy, apoptosis and necrosis signature gene was marked with blue, yellow and red respectively. Green dots in the circle represent protective factors of prognosis and black dots in the circle represent risk factors of prognosis. The lines linking signature genes showed their interactions, and thickness showed the correlation strength between genes. Negative correlation was marked with blue and positive correlation with red

To determine the prognostic signature of each cell death pathway, higher expression of genes in the training set (TCGA) were evaluated for prognostic correlation with OS, and various gene combinations were tested. Finally, the 4-gene apoptosis signature, 8-gene autophagy signature, and 4-gene necrosis signature displayed prognostic association in GBM (Table 1) and the combined 16-gene cell death index (CDI) was generated (Fig. 1A). The interaction between CDI signature genes in GBM is displayed in Fig. 1B.

**Table 1:** The prognostically significant gene signature within apoptosis, autophagy and necrosis in Glioblastoma

Cell Death Process	Gene	Gene ID	Gene (Full Name)
Apoptosis	BID	637	BH3 interacting domain death agonist
	CFLAR	8837	CASP8 and FADD like apoptosis regulator
	CHP1	11,261	Calcineurin like EF-hand protein 1
	PRKAR1B	5575	Protein kinase cAMP-dependent type I regulatory subunit beta
Autophagy	SREBF1	6720	Sterol regulatory element binding transcription factor 1
	SERPINA1	5265	Serpin family A member 1
	PRKAG2	51,422	Protein kinase AMP-activated non-catalytic subunit gamma 2
	PRKAB2	5565	Protein kinase AMP-activated non-catalytic subunit beta 2
	MET	4233	MET proto-oncogene, receptor tyrosine kinase
	MAPK3	5595	Mitogen-activated protein kinase 3
	LAMTOR3	8649	Late endosomal/lysosomal adaptor, MAPK and MTOR activator 3
	EEF1A2	1917	Eukaryotic translation elongation factor 1 alpha 2
Necrosis	CASP3	836	Caspase 3
	NOL3	8996	Nucleolar protein 3
	TRAF3	7187	TNF receptor associated factor 3
	TRAP1	10,131	TNF receptor associated protein 1

### RNA-Seq Analysis of Patients in TCGA

**Fig. 2:**
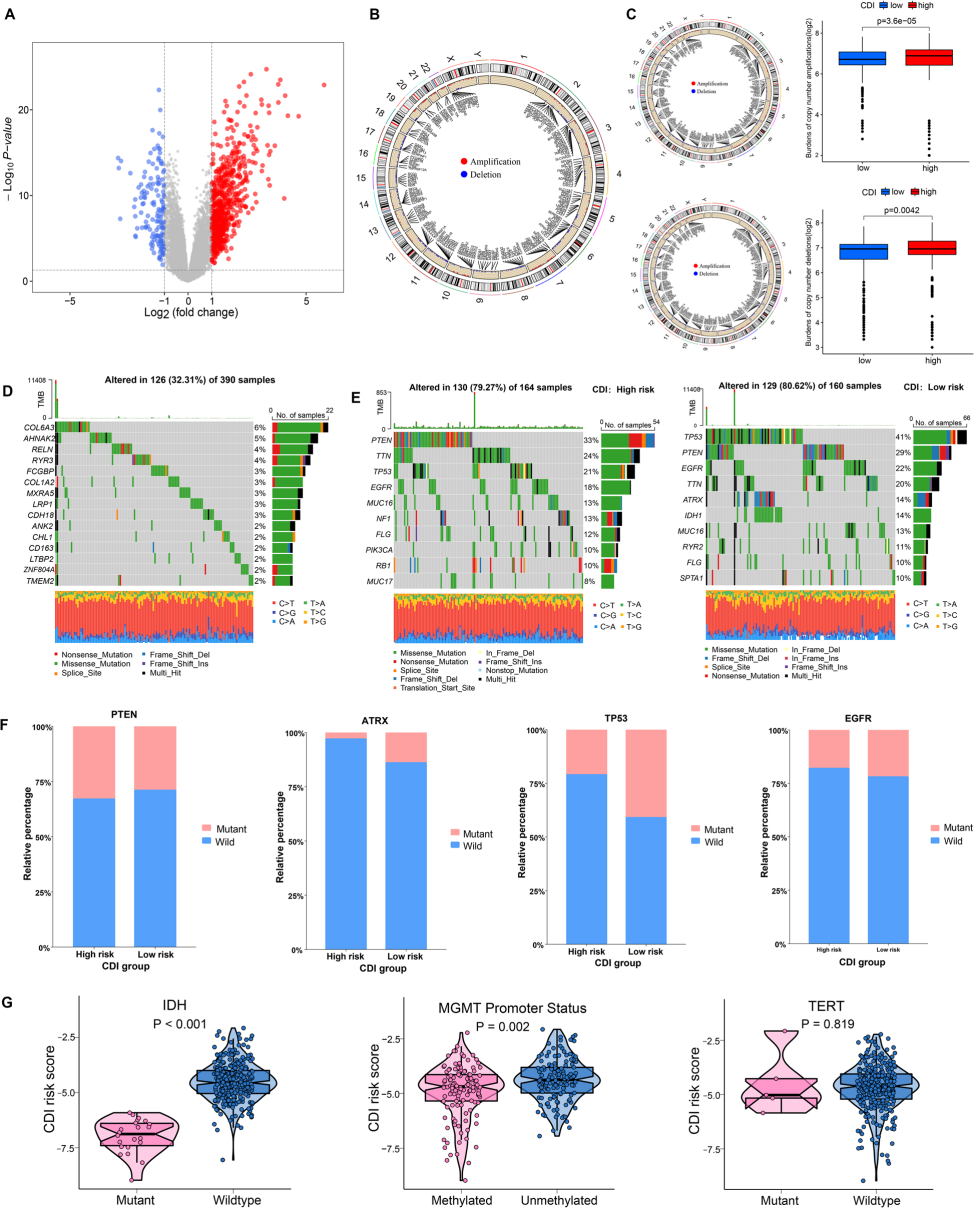
**A**) Volcano plot showing the differential expression of genes between high CDI and low CDI patients (*p* < 0.05, |log2 fold-change|> 1). **B**) The differential analysis of copy number variations between two groups was visualized by Circos plot, which revealed that compared with the low CDI group, 225 (35.3%) genes were significantly amplified, and 309 (48.4%) were significantly deleted in the high CDI group. Red dots represented amplifications and blue dots represented deletions. **C**) Left panel: Circos plots of each risk group revealing the amplifications and deletions of chromosomes. Right panel: Boxplots inhibited more burdens of copy number amplifications and deletions in high CDI group. **D**) Waterfall plots of 15 most frequently mutated DEGs which were altered in 126 GBM samples. **E**) Waterfall plots showed the top 10 mutated in high risk and low risk group. **F**) The proportion of mutation status of PTEN, ATRX, TP53 and EGFR in the two groups. **G**) Violin plots of CDI risk score in individual samples of GBM patients, stratified by IDH, MGMT promoter and TERT mutation status

Seven hundred sixty-nine differentially expressed genes were identified and 621 of them were upregulated at > onefold in high CDI group compared to the low CDI group (Table S4). In the low CDI group, 148 genes were upregulated at > onefold compared to the high CDI group (Table S5). The volcano plot of differentially expressed genes between two groups is illustrated in Fig. 2A.

### High CDI patients possessed a higher CNA burden and lower TMB

CNA and somatic mutation analyses were carried out to investigate the genomic variations in the two CDI risk groups. The differential analysis of CNA between the two groups showed that, compared with the low CDI group, 225 (35.3%) genes were significantly amplified, and 309 (48.4%) genes had deletion variation in the high CDI group (Fig. 2B). Moreover, the boxplots exhibited more copy number amplifications(*p* = 3.6e-05) and deletions(*p* = 0.042) burdens in the high CDI group (Fig. 2C). Somatic mutation analysis revealed that 31 out of the 769 DEGs (4.0%) had a mutation frequency > 1%, and most (83.9%, 26/31) of them were upregulated in the high CDI group. The top 15 most frequently mutated DEGs altered in the 126 GBM samples were visualized by oncoplot and illustrated in Fig. 2D. Somatic mutation analysis of two CDI groups separately uncovered that each risk group had distinct top mutated genes (Fig. 2E). In the low CDI group, TP53 (41%) was most frequently mutated, higher than that in the high CDI group, in which PTEN (33%) was most frequently mutated (Fig. 2F). Figure 2G demonstrated that the CDI risk score was significantly higher in IDH-wide-type samples than in IDH-mutant samples (*P* < 0.001) and patients with MGMT promoter methylation (*P* = 0.002) showed significantly lower CDI risk scores. All these findings might uncover the underlying differences in response to immunotherapy between the two groups.

### Associations between CDI and clinical features

We calculated the CDI of 518 GBM patients and ranked them from low to high to analyze the associations between the CDI and clinical features. The demographics and follow-up data of the patients in the high CDI and low CDI groups were compared and presented in Table 2. In the TCGA cohort, univariate and multivariate analyses demonstrated that the high CDI group was significantly associated with OS (HR = 2.850, 95%CI:1.981–4.100, *P* < 0.001) (Table 3) and PFS (HR 2.099, 95%CI:1.541–2.858, *P* < 0.001) (Table S6), suggesting that the CDI high risk could independently predict the OS and PFS of GBM patients. In addition, radiotherapy was a favorable factor for patients’ survival (OS, HR = 0.224, 95% CI:0.125–0.403, *P* < 0.001; PFS, HR = 0.420, 95% CI:0.299–0.589, *P* < 0.001).

**Table 2:** Demographics and clinicopathological features of GBM patients in the TCGA and CGGA cohort

Variables	TCGA cohort			CGGA cohort		
	**Total (*n* = 518)**	**CDI low risk (*n* = 248)**	**CDI high risk (*n* = 270)**	**Total (*n* = 137)**	**CDI low risk (*n* = 66)**	**CDI high risk (*n* = 71)**
**Age (years)**	57.54 ± 14.60	55.30 ± 15.94	59.60 ± 12.95	46.61 ± 12.56	42.88 ± 11.30	50.08 ± 12.76
**Gender**						
Male	314	154	160	87	37	50
Female	204	94	110	50	29	21
**Surgery**						
Tumor resection	451	211	240	NA	NA	NA
Biopsy only	65	36	29	NA	NA	NA
NA	2	1	1	NA	NA	NA
**Pretreatment KPS**						
≥ 80	286	151	135	NA	NA	NA
< 80	101	41	60	NA	NA	NA
NA	131	56	75	NA	NA	NA
**Radiotherapy**						
Yes	402	207	195	100	47	53
No	94	33	61	32	19	13
NA	22	8	14	5	0	5
**TMZ chemotherapy**						
Yes	298	154	144	99	51	48
No	197	85	112	34	15	19
NA	23	9	14	4	0	4
**Standard chemoradiotherapy**						
Yes	199	101	98	NA	NA	NA
No	297	139	158	NA	NA	NA
NA	22	8	14	NA	NA	NA
**TCGA subtype**						
Classical	143	57	86	NA	NA	NA
Mesenchymal	152	40	112	NA	NA	NA
Neural	88	51	37	NA	NA	NA
Proneural	135	100	35	NA	NA	NA
**G-CIMPstatus**						
G-CIMP	45	44	1	NA	NA	NA
Non G-CIMP	473	204	269	NA	NA	NA
**IDH status**						
Mutant	30	29	1	39	37	2
Wild type	368	179	189	98	29	69
NA	120	40	80	0	0	0
**MGMT promoter status**						
Methylated	170	94	76	65	41	24
Unmethylated	177	69	108	70	24	46
NA	171	85	86	2	1	1
**1p/19q status**						
Codeletion	NA	NA	NA	7	7	0
Non-codeletion	NA	NA	NA	127	58	69
NA	NA	NA	NA	3	1	2
**Overall Survival (OS)**						
> 3 years	48	44	4	17	11	6
≤ 3 years	470	204	266	120	55	65
**OS status**						
Deceased	441	203	238	124	59	65
Living	77	45	32	13	7	6
**Progression-Free Survival (PFS)**						
> 3 years	24	22	2	NA	NA	NA
≤ 3 years	494	226	268	NA	NA	NA
**PFS status**						
Progression	442	201	241	NA	NA	NA
No progression	76	47	29	NA	NA	NA

**Table 3:** Univariate and multivariate cox proportional hazards analysis of clinicopathological variables based on overall survival (OS) in the TCGA GBM training cohort and CGGA validation cohort

Variables	TCGA training cohort(*n* = 518)						CGGA validation cohort(*n* = 137)					
	Univariate analysis			Multivariate analysis			Univariate analysis			Multivariate analysis		
	HR	95% CI	*p*.value	HR	95% CI	*p*.value	HR	95% CI	*p*.value	HR	95% CI	*p*.value
Age	1.032	1.025–1.040	1.98E-17	**1.019**	**1.005–1.033**	**0.007**	1.010	0.992–1.02	3.62E-01			
Gender	1.160	0.956–1.408	1.32E-01				1.220	0.84–1.78	2.94E-01			
Surgery	1.166	0.884–1.538	2.76E-01				NA			NA		
KPS	0.503	0.389–0.651	1.79E-07	0.981	0.669–1.439	0.922	NA			NA		
TMZ chemotherapy	0.578	0.476–0.703	3.82E-08	0.790	0.481–1.297	0.352	0.490	0.326–0.737	6.06E-04	**0.494**	**0.328–0.743**	**7.15E-04**
Radiotherapy	0.281	0.220–0.359	1.58E-24	**0.224**	**0.125–0.403**	**0.000**	0.871	0.568–1.34	5.26E-01			
Standard chemoradiation	0.815	0.667–0.997	4.62E-02	1.017	0.696–1.486	0.932	NA			NA		
G-CIMPstatus	0.345	0.238–0.502	2.37E-08	0.271	0.035–2.101	0.211	NA			NA		
TCGA Subtype	0.926	0.855–1.004	6.08E-02				NA			NA		
IDH mutation status	0.362	0.226–0.577	2.03E-05	3.435	0.420–28.099	0.250	1.000	0.681–1.48	9.87E-01			
MGMT promoter methylation status	0.674	0.529–0.858	1.36E-03	0.869	0.625–1.209	0.404	0.898	0.628–1.28	5.53E-01			
1p/19q codeletion status	NA			NA		NA	0.783	0.363–1.69	5.32E-01			
CDI high risk	2.386	1.949–2.920	3.27E-17	**2.850**	**1.981–4.100**	**0.000**	1.520	1.06–2.17	2.31E-02	**1.498**	**1.037–2.166**	**3.15E-02**

*NA* not available, *HR* hazard ratio, *CI* confidence interval, *KPS* Karnofsky performance score, *TMZ* temozolomide; All statistical tests were two sided. Bold type means *P* < 0.05

**Fig. 3:**
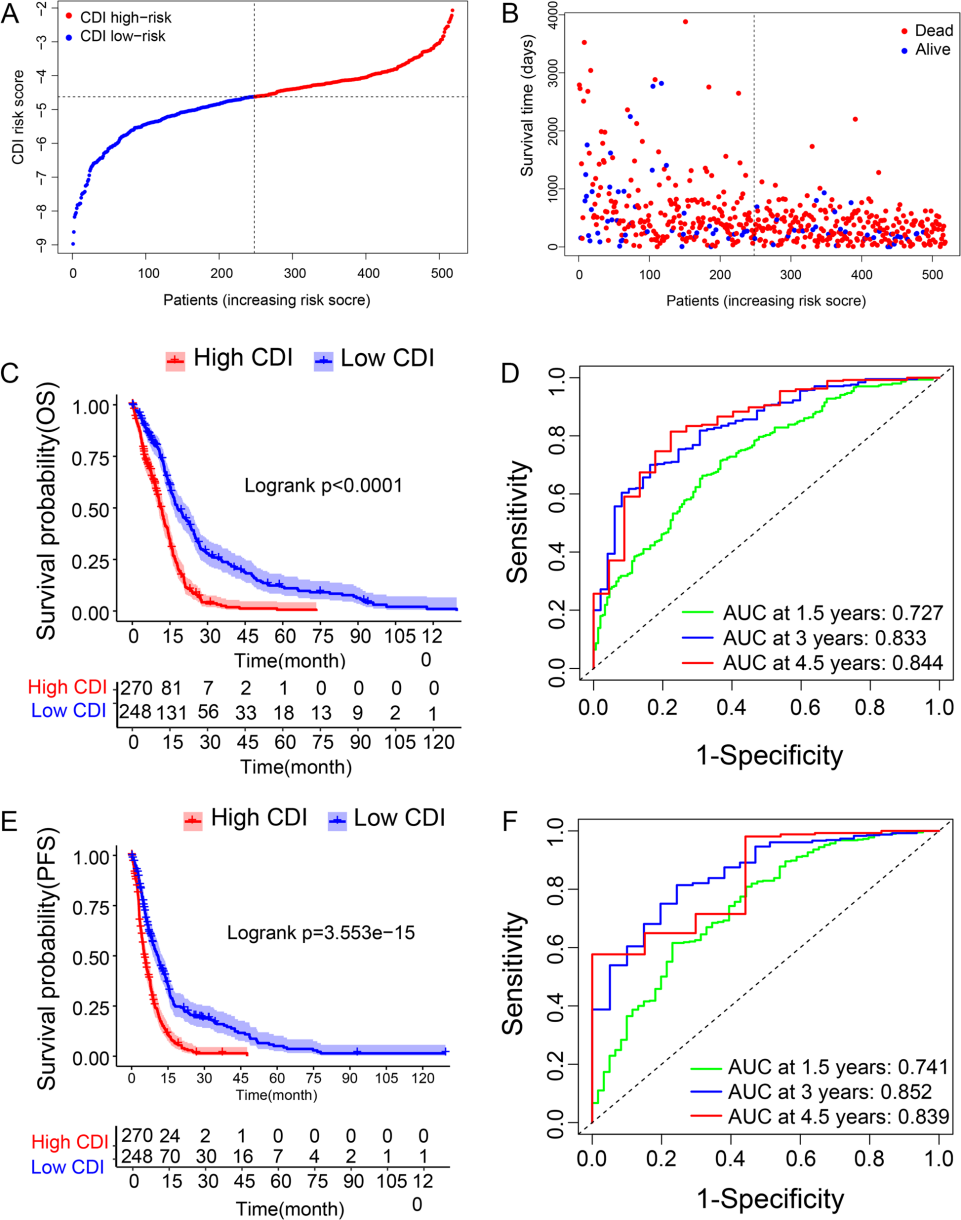
Distribution of the CDI in TCGA training cohort. **A**) Classification of patients into different risk groups based on CDI. **B**) Distribution of patients’ survival time and status. **C**) Kaplan–Meier survival analysis suggested that patients’ OS were significantly different between high CDI and low CDI group. **D**) The prognostic performance of CDI demonstrated by ROC curves for predicting the 1.5-, 3.0-, and 4.5- year OS rates. **E**) Kaplan–Meier survival analysis suggested that patients’ PFS were significantly different between high CDI and low CDI group. **F**) The prognostic performance of CDI demonstrated by ROC curves for predicting the 1.5-, 3.0-, and 4.5- year PFS rates

As illustrated in Fig. 3, patients were assigned into either the high CDI group or the low CDI group according to their median risk score (Fig. 3A). With the risk score increasing, the number of alive patients reduced (Fig. 3B). Kaplan–Meier analyses exhibited that patients with a high CDI had significantly worse OS and PFS than those with a low CDI (Fig. 3C, P < 0.0001; Fig. 3E, P < 0.0001). The predicted efficiencies of CDI were evaluated by ROC curves (Fig. 3D and Fig. 3F). The results illustrated that the AUCs of CDI for predicting the 1.5-, 3- and 4.5-year OS were 0.727, 0.833 and 0.844, respectively.

### Validation of CDI

**Fig. 4:**
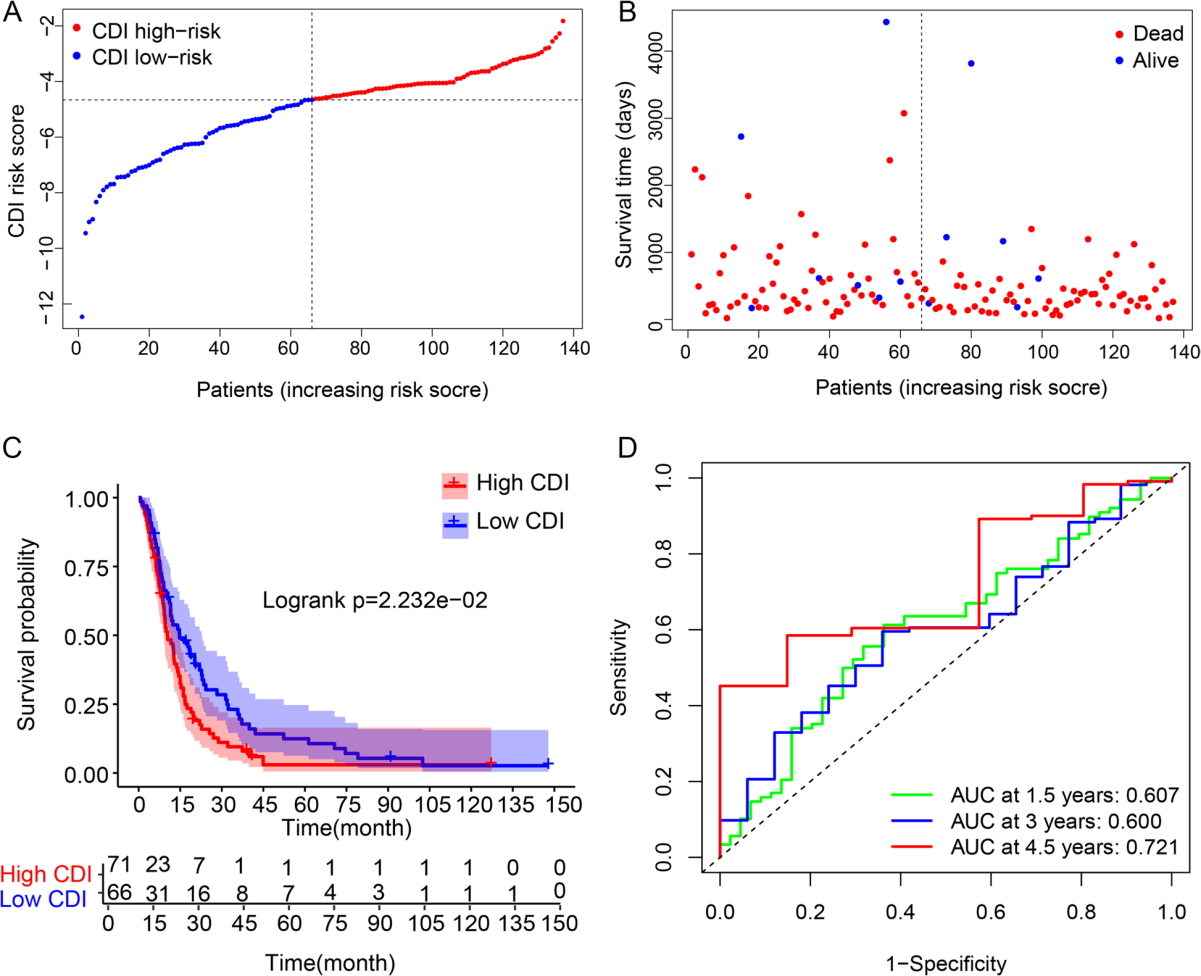
Distribution of the CDI in CGGA validation cohort. **A**) Classification of patients into different risk groups based on the CDI. **B**) distribution of patients’ survival time and status. **C**) Kaplan–Meier survival analysis suggested that patients’ OS were significantly different between high CDI and low CDI group. **D**) The prognostic performance of CDI demonstrated by ROC curves for predicting the 1.5-, 3.0-, and 4.5- year OS rates

One hundred thirty-seven GBM patients in the CGGA database were enrolled as validation data set for assessing the signature performance. The CDI risk score of the validation cohort patients was then calculated according to the risk score formula and subsequently allocated into either the high CDI or the low CDI group (Fig. 4A). The demographics and clinicopathological data of the patients in the two groups were compared and presented in Table 2. Univariable and multivariable Cox analyses found that the high CDI group was significantly associated with OS (HR = 1.498, 95%CI:1.037–2.166, *P* < 0.05) (Table 3). Consistent with the findings in TCGA, the Kaplan–Meier survival curves demonstrated that high CDI patients had a poorer prognosis than those with a low CDI (Fig. 4C, *P* < 0.0001). The AUCs of ROC curves for predicting the 1.5-, 3.0- and 4.5-year survival of GBM in the dataset were 0.607, 0.600, and 0.721, respectively (Fig. 4D). Concomitantly, the OS and alive patients deceased with an increase in risk scores (Fig. 4B). These results implied a satisfactory performance of CDI for survival prediction in GBM patients.

### Cytokine Gene Expression Analysis

**Fig. 5:**
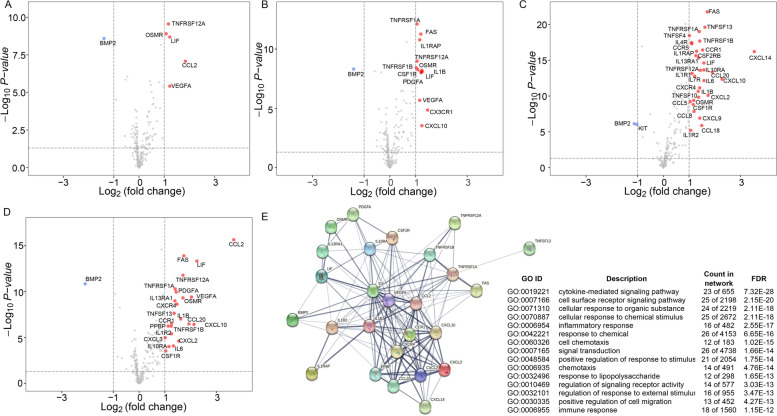
Volcano plot showing the differential expression of cytokines (*n* = 265 genes) between patients in the high and low significant gene expression of individual cell death pathway (*p* < 0.05): **A**) apoptosis, **B**) autophagy, **C**) necrosis, **D**) CDI. **E**) Gene Ontology (GO) functional enrichment of genes with higher expression in high CDI group

In the autophagy group, TNFRSF12A, OSMR, LIF, CCL2 and VEGFA displayed higher expression levels in the high risk group, whereas BMP2 showed a higher expression level in the low risk group (Fig. 5B). Analyses conducted on the apoptosis and necrosis groups were depicted in Fig. 5A and 5C. In the CDI group, the expression level of CCL2, LIF, FAS, IL1B, CXCL10, CCL20, TNFSF13, IL6, IL1R2, IL10RA, and IL13RA1, among others, were higher in high CDI patients whereas the BMP2 expression level was higher in the low CDI patients (Fig. 5D). Furthermore, functional enrichment analysis showed that the high CDI group seemed to have a higher inflammatory cytokines proportion than the low CDI group (Fig. 5E).

### Immune cell analysis

**Fig. 6:**
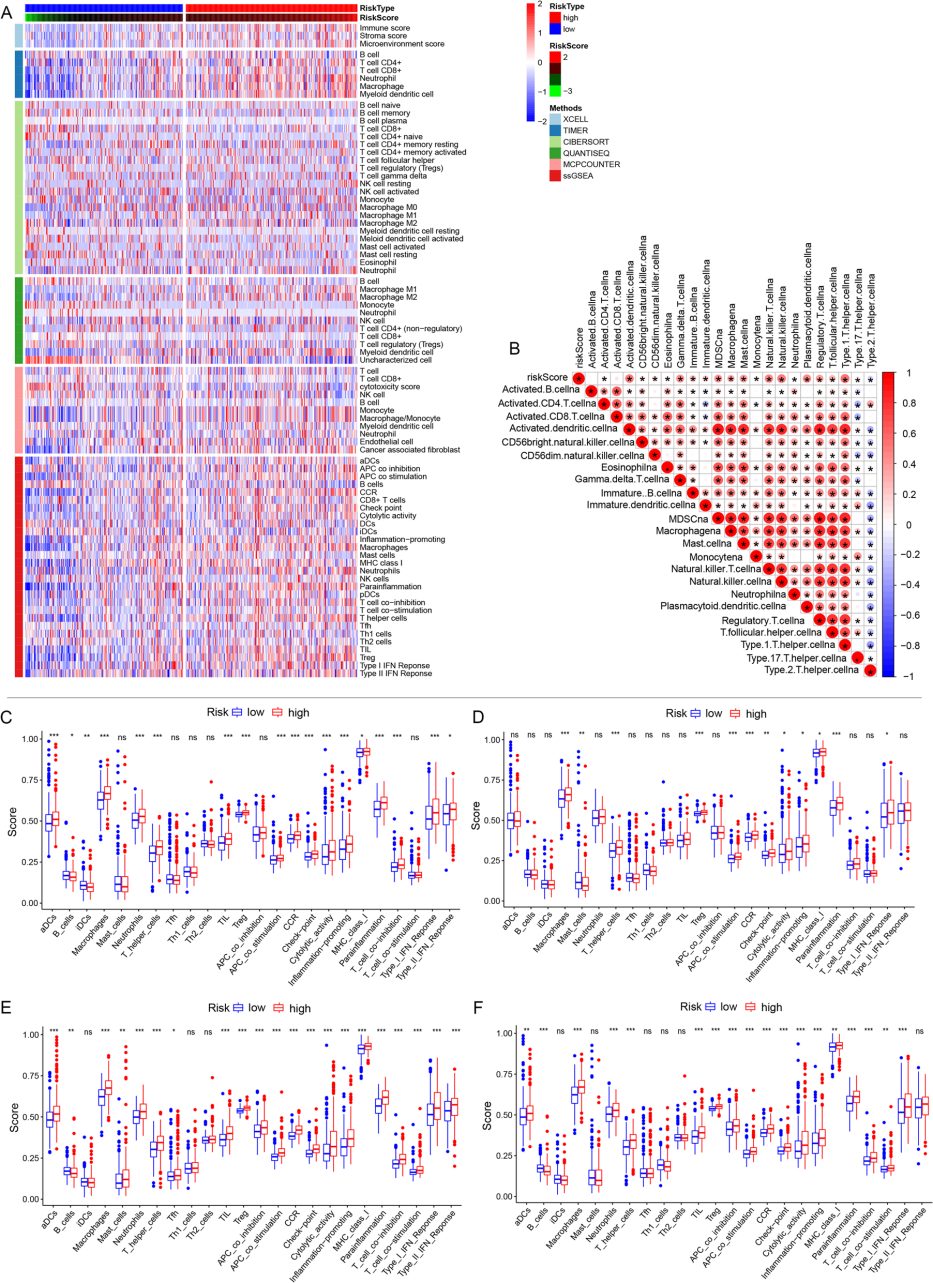
**A**) Heatmap for immune responses based on XCELL, TIMER algorithms, CIBERSORT, quanTIseq, MCPcounter and ssGSEA among high and low risk group. **B**) Correlations between CDI riskscore and the immune cells using Spearman analysis. Negative correlation was marked with blue and positive correlation with red. Immune cell distribution and immune function in patients with the high and low significant gene expression of individual cell death pathway: **C**) apoptosis, **D**) autophagy, **E**) necrosis, **F**) CDI

Figure 6A displays the heatmap of immune responses between low CDI and high CDI groups, based on CIBERSORT, xCell, MCP-counter, quanTIseq, ssGSEA and TIMER algorithms. The results established that the high CDI group exhibited higher immune scores, indicating a significantly increased immune cell infiltration. To estimate the correlations between the CDI and immune cells, spearman analysis was performed, and the results are illustrated in Fig. 6B. Correlation analysis based on the ssGSEA of the TCGA dataset showed that higher Treg, T helper cell and macrophages infiltration correlated with the high risk group of all the three cell death pathways (Fig. 6C, 6D and 6E). Notably, neutrophils and aDCs infiltration were higher in patients with high necrosis, apoptosis, and CDI groups. In contrast, B cells were enriched in patients with low necrosis, apoptosis, and CDI. Furthermore, the ssGSEA algorithm demonstrated a significant difference between the low CDI and high CDI groups in T cell functions, including checkpoint (inhibition), cytolysis, CCR, regulation of inflammation, co-stimulation of T cell, co-inhibition of T cell, and type I INF response, as illustrated in Fig. 6F. Altogether, these results imply that CDI may present a novel understanding of the immune response in GBM.

### CDI correlated with Key Genes of ICB Therapy in GBM

**Fig. 7:**
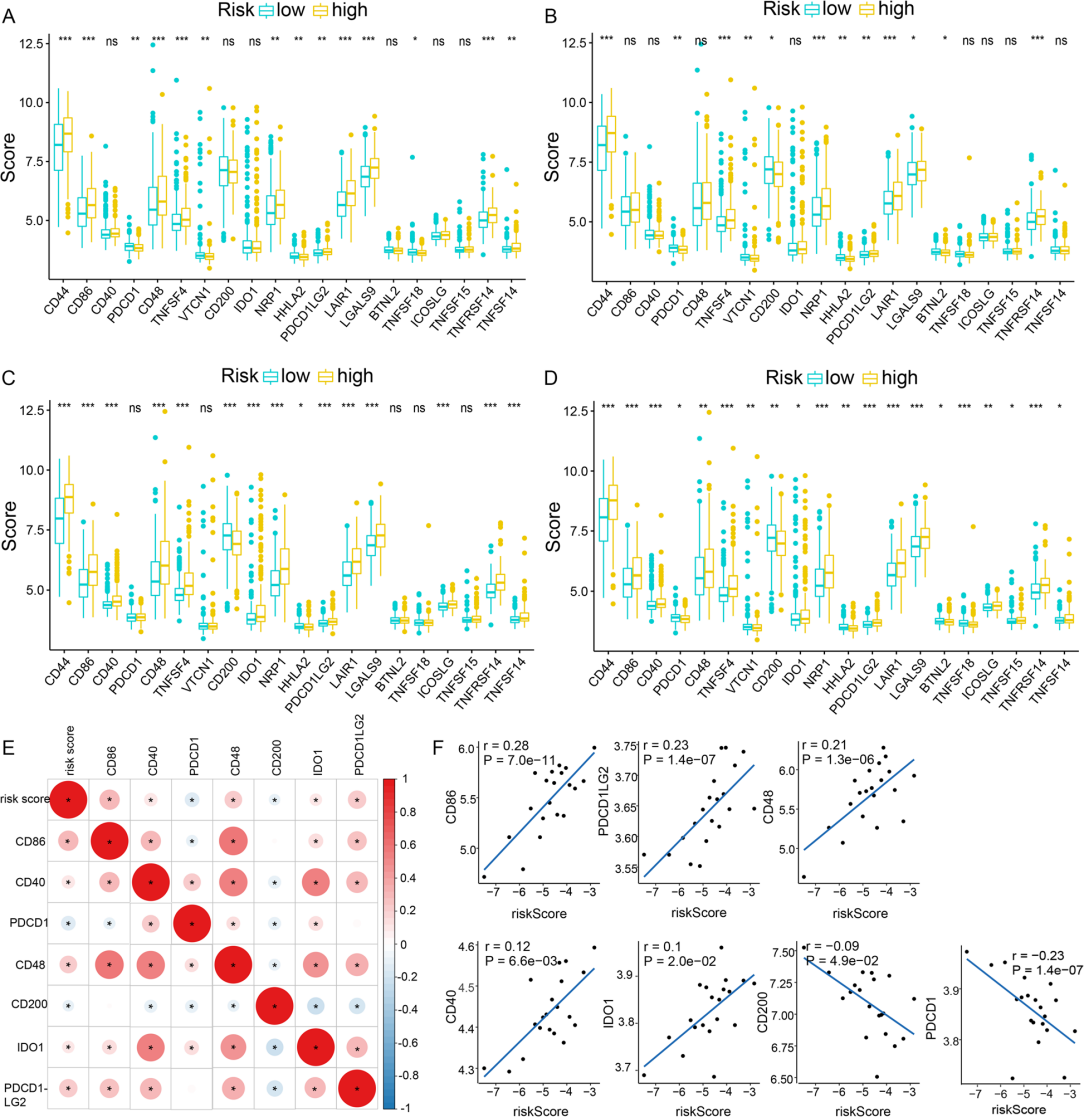
Comparison of immune checkpoint blockade–related genes expression levels in patients with the high and low significant gene expression of individual cell death pathway: **A**) apoptosis; **B**) autophagy, **C**) necrosis, **D**) CDI. **E**) Association analyses between immune checkpoint inhibitors CD86, CD40, PDCD1, CD48, CD200, IDO1 and PDCD1LG2 and CDI. **F**) Correlation plot showing the association between CDI risk model and CD86, PDCD1LG2, CD48, CD40, IDO1, CD200 and PDCD1

Correlation analysis showed that the expression levels of 20 (i.e., PDCD1, IDO1, etc.) ICB-related genes were significantly different between high CDI and low CDI groups (Fig. 7D). Similar analyses were also performed in the other three classically known cell death pathways (Fig. 7A, 7B and 7C). We singled out seven key ICI genes (CD86, CD40, PDCD1, CD48, CD200, IDO1, and PDCD1LG2) for further research. To investigate the potential effect of the CDI in the ICB therapy of GBM, the correlation analyses were performed between the expression level of the ICB key genes and the CDI risk scores (Fig. 7E). The result revealed that the CDI risk score had close relationship with CD86 (*r* = 0.28; *p* = 7.0e − 11), PDCD1LG2 (*r* = 0.23; *p* = 1.4e − 07), CD48 (*r* = 0.21; *p* = 1.3e − 06), and PDCD1(*r* = -0.23; *p* = 1.4e − 07) (Fig. 7F), indicating CDI might have a nonnegligible role in the outcome prediction of ICB treatment in GBM.

### Enrichment analysis

**Fig. 8:**
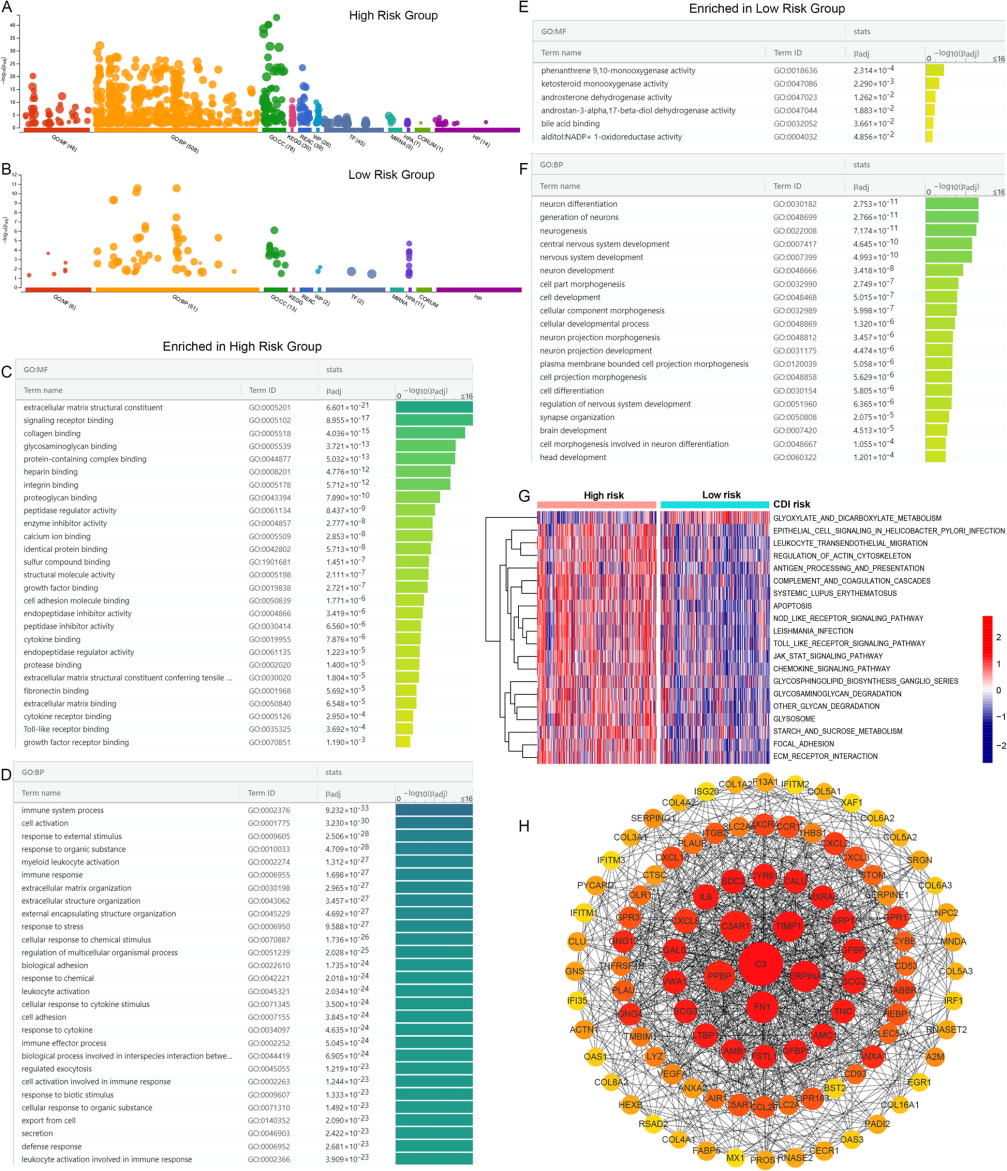
Functional enrichment analysis of highly expressed genes (log2 fold-change > 1) in the **A**) high risk group; and **B**) low risk group. Pathways enriched in high and low CDI groups: Molecular Function (GO: MF) and Biological Process (GO: BP) of the (**C**, **D**) high CDI group and (**E**, **F**) the low CDI group. **G**) Heatmap illustrated the enrichment scores of 20 differentially enriched molecular pathways evaluated by GSVA analysis between low CDI and high CDI patients. Red represented high enrichment scores, and blue represented low enrichment scores. **H**) PPI network of hubba DEGs obtained from the DEGs network

Functional enrichment analysis of differential gene expressions between high CDI and low CDI groups identified 769 genes based on an inclusion filter of > onefold for the DEGs. 621 genes of them were upregulated in high CDI group, while 148 genes of them were upregulated in the low CDI group (Fig. 8A and 8B). Patients in high CDI group had significant immune-related pathways, whereas the patients in low CDI group lacked enrichment in immune-related pathways. The enriched Gene Ontology terms in high CDI group were receptor ligand activity, signaling, cytokine, and chemokine activity (Fig. 8C and 8D). Conversely, the enriched Gene Ontology terms in low CDI group were predominated by transmembrane transporters and gated channel activity (Fig. 8E and 8F).

Potential molecular pathways and underlying mechanisms related to the CDI of GBM were investigated by performing GSVA. The results identified that 19 pathways were positively correlated with the high CDI group, while 1 pathway was positively correlated with the low CDI group (Fig. 8G). High CDI patients mainly correlated with toll-like receptors signaling, JAK-STAT signaling, chemokine signaling pathway, complement and coagulation cascades, ECM receptor interaction, and others, whereas the low CDI group mainly correlated with glyoxylate and dicarboxylate metabolism.

The PPI network of the 769 overlapping DEGs was selected from the STRING database. The top 100 hub proteins were mined using Cyto-Hubba, and the PPI network was then performed and visualized in the Cytoscape software (Fig. 8H). The network suggested strong interactions among these hub genes.

### Potential compounds targeting the two CDI groups

CMap analysis was performed to investigate potential drugs targeting the two CDI groups of GBM (Table S7, Table S8, and Figure S1). Compounds with a score less than -90.0 or higher than 90.0 were selected, the predicted drugs are listed in Table 4. It was determined that nine compounds targeting 7 molecular pathways had a score of less than -90 and were potential inhibitors for high CDI patients. On the other hand, 5 pathways targeted by 7 compounds with a score higher than 90 were considered potential inhibitors for low CDI patients.

**Table 4:** Significant therapeutic drugs predicted by Cmap

Drug Name	Mechanism of Action	Target	Score
Tipifarnib	Farnesyltransferase inhibitor	FNTA, FNTB	-96.1
Tofacitinib	JAK inhibitor	JAK3, JAK1, JAK2, CYP2C19, TYK2	-95.81
GSK-1070916	Aurora kinase inhibitor	AURKB, AURKC, AURKA, CYP2D6, CYP3A4	-94.93
Mestranol	Estrogen receptor agonist	ESR1	-94.48
Ruxolitinib	JAK inhibitor	JAK1, JAK2, TYK2, JAK3	-93.19
XMD-1150	Leucine rich repeat kinase inhibitor	LRRK2	-92.64
TPCA-1	IKK inhibitor	IKBKB	-91.9
PPT	Estrogen receptor agonist	ESR1	-91.84
7,4'-dihydroxyflavone	Opioid receptor antagonist	CYP19A1	-91.29
Phorbol-12-myristate-13-acetate	PKC activator	CD4, KCNT2, PRKCA, TRPV4	90.49
BX-795	IKK inhibitor	PDPK1, CDK2, CHEK1, GSK3B, IKBKE, KDR, PDK1, TBK1	92.53
Arecaidine	Acetylcholine receptor agonist	CHRM1, CHRM2, CHRM3, CHRM4	92.67
NSC-94258	Antineoplastic	AKR1B1, CYP19A1, HSD17B1	93.08
Scoulerine	Adrenergic receptor antagonist, GABA receptor antagonist, Serotonin receptor antagonist	ADRA1D, ADRA2A, GABRA1	95.29
Prostratin	PKC activator	PRKCA, PRKCB, PRKCD, PRKCE, PRKCG, PRKCH, PRKCQ	95.81
Ingenol	PKC activator	PRKCD, PRKCE	96.05

## Discussion

GBM is the most common primary intracranial tumor with high malignancy and poor outcomes. Relevant molecular biomarker models for predicting the prognosis of GBM, such as hypoxia, ferroptosis-related gene, and stem cell signatures, have been established to improve patients’ survival and develop novel individualized treatments [55–57]. However, their accuracy and predictive abilities remain limited, and most of them were developed based on single transcriptomics data with inadequate focus on biological pathways. Autophagy, apoptosis and necrosis represent three significant biological hallmarks of tumors that are valuable in predicting GBM patients’ prognosis. In this study, we constructed a combined gene signature involved in autophagy, apoptosis, and necrosis, and evaluated its prognostic effect and correlation with immune cells and mediators.

We first integrated the genomic information of 518 GBM patients from the TCGA database to comprehensively evaluate the prognostic of genes involved in the three classically known cell death pathways and identified a novel 16-gene CDI signature. Among these significant genes of the signature, BID, CHP1, MAPK3, PRKAB2, PRKAG2 and TRAP1, were favorable factors for GBM patients’ survival in this study. BID is a pro-apoptotic member of the Bcl-2 protein family and could promote Ca2 + -induced neuronal injury [58]. Inhibition of BID during acute endoplasmic reticulum stress may protect against cell death [59]. In addition, studies reported that BID was associated with the prognosis of immunoglobulin A nephropathy [60]. TRAP1 which affects the mitochondrial protein quality control system and mitochondrial metabolism, has multiple functions in mitochondria. Researchers found that suppressing the function of TRAP1 would benefit temozolomide therapy in GBM in vitro, revealed great potential and practical value in GBM treatment. [61]. CHP1, MAPK3, PRKAB2 and PRKAG2, these four genes, were rarely focused on. Other ten genes of the CDI gene signature, including CFLAR, PRKAR1B, EEF1A2, LAMTOR3, MET, SERPINA1, SREBF1, CASP3, NOL3, TRAF3, were risk factors for GBM patients’ survival. EEF1A2 is a translation factor selectively expressed by heart, skeletal muscle, nervous system and some specialized cells. It is a putative oncogene highly expressed in ovarian cancer [62]. Studies have shown its negative prognostic role in breast cancer, non-small cell lung cancer and gastric cancer [63–66]. MET plays a well-defined role as a selectable oncogenic driver of tumor proliferation. Preclinical studies found that inhibition of MET could reduce cell survival, local invasion and metastasis to distant sites [67]. CASP3 plays an important role in the development of the brain. Moderate active CASP3 levels were found in human GBM samples and down regulation of CASP3 may inhibit the migration GBM cells, suggesting that CASP3 inhibition may serve as a novel therapeutic strategy for GBM [68]. NOL3 protects against oxidative stress-induced cell death. Researches showed a tumor suppressor role of NOL3 in the pathogenesis of myeloid malignancies [69]. LAMTOR3 and SERPINA1 both showed prognostic effect in tumors [70, 71]. Compared with the previous autophagy-related gene signatures for GBM, the 8 autophagy-related genes included in CDI were all novel [72–74]. In addition, their data source and bioinformatic processes are different. Our findings could propose several potential targets for glioblastoma therapy. As the first prognostic model with three cell death pathway-related genes for GBM patients, the CDI signature can simultaneously reflect the tumor’s cell death characteristics and is convenient for clinical use.

We classified patients into two groups based on their CDI, and analyzed the correlation between CDI subtypes and clinical features. The results indicated that low CDI patients had longer OS and PFS. Moreover, the correlation between CDI and clinicopathological parameters, as well as survival outcomes, suggests that CDI has an effective prognostic prediction in GBM patients. Afterward, the CDI signature was verified in the CGGA cohort patients. ROC curves and survival analysis revealed an efficient performance of CDI.

Further in this study, we explored the correlation of CDI with the TME cell infiltration and ICI in the prognosis of GBM. GBM is one of the most immunologically “cold” tumors, which presents an unsatisfactory treatment effect with immunotherapy [75]. Although the results so far are mostly disappointing, a large number of clinical trials indicated that immunotherapy remains conceptually promising for GBM treatment [76–78]. Previous studies revealed that the poor results of immunotherapy were mainly due to the profound immunosuppressive characteristic of GBM and a TME that is challenging for immune cells [26]. Profiling the tumor immune microenvironment has lately been regarded as one of the future breakthroughs to improve immunotherapy of GBM [79, 80]. Herein, neutrophils, macrophages, T helper cells, Tregs, TIL, and aDCs were enriched in patients with a high CDI. The CDI risk score was significantly correlated with immune cell infiltration (i.e., neutrophils, macrophages, T helper cells, Tregs, TIL, and aDCs). Besides, the ssGSEA analysis also pointed that the infiltrating immune cells (i.e., neutrophils, macrophages, T helper cells, Tregs, TIL, and aDCs) were remarkably increased, and immune signatures (i.e., checkpoint, cytolytic, CCR, regulation of inflammation, co-stimulation of T cell, co-inhibition of T cell, and type I INF response) were significantly activated when the risk score was elevated, indicating that the CDI signature possesses an unneglectable role in the TME of GBM. The higher neutrophils and macrophages infiltration in high CDI risk patients reflected high inflammation and local immune dysfunction in TME [81]. In fact, the presence of macrophages has been approved to be a negative predictor for survival in high-grade gliomas murine models and it could impair antitumor immunological functions of TME in glioblastoma [82, 83]. Effector T cell infiltration in the tumor has been identified positively correlated with patients’ survival whereas the Treg fraction increase in GBM patients implied a deficit of patient effector T-cell responsiveness, suggesting that higher Tregs might be related with poor survival of patients [84, 85]. All the above studies suggest correlations between the CDI and TME. Therefore, targeting specific GBM patients based on CDI selection may help immunotherapy achieve a superior therapeutic effect.

With the development of ICB treatment, ICIs have greatly affected the cancer treatment scheme [86, 87]. Indeed, ICB treatment has brought a novel field for GBM patients’ clinical management [88, 89]. Even though current clinical trials of ICB treatment in GBM failed, demonstrating appropriate patients, benefiting from ICB treatment, through various biomarkers, still appears promising in GBM treatment. In this study, the CDI signature was negatively associated with the ICB treatment key target genes(i.e. PDCD1 and CD200), and the expression level of some ICB-related genes ( i.e. PDCD1LG2,CD86, CD48, CD40, and IDO1) elevated significantly with an increase of the CDI risk score. The activation of IDO is involved in tumorigenesis by helping tumor cells evade immune surveillance [90]. In addition, studies have found that high IDO1 expression was associated with the poor prognosis of GBM [91]. Therefore, ICB therapy targeting patients with high IDO1 might achieve superior treatment effect. Besides, our data also revealed that low CDI risk patients were more likely to have a higher tumor mutation burden. Researchers construed that the low TMB in GBM was a fundamental cause for its failure in immunotherapy except for the immunosuppressive microenvironment [92, 93]. Thus, low CDI patients with high TMB were speculated to show a satisfactory therapeutic effect with ICB treatment. Overall, our findings indicated that CDI might well predict the clinical outcome of ICB therapy in GBM patients.

CMap analysis was performed to find potential compounds targeting genes related to CDI, enabling more practical results. In the case of the high CDI group, ruxolitinib was uncovered as one of the candidate drugs. Ruxolitinib was recently found to prevent glioblastoma invasion and tumorigenesis by inhibiting the IFN-induced JAK/STAT signaling pathway [94, 95]. Besides, it has also been proposed for use in patients with chronic neutrophilic leukemia for its safety and efficacy in inhibiting JAK1/2 [96]. For the low CDI group, 3 out of 7 compounds with a score higher than 90 were PKC activators, consistent with a recent study that confirmed PKC as a suitable druggable target to treat recurrent GBM [97]. The above researches demonstrated the reliability of drug screening in our study and the feasibility to applicate in GBM treatment. To further authenticate the therapeutic value of these compounds, more studies are warranted.

Although the results of our study indicate that CDI can serve as an effective prognostic signature for GBM patients, there are also some limitations. First, our research is mainly based on integrating the genomic data from public datasets to comprehensively assess the role of CDI with the TME cell-infiltrating characteristics in GBM and predicted potential compounds targeting patients with different CDI scores. Verification by cell and animal experiments can undoubtedly make our results more reliable. But due to our limited laboratory and samples, it is extremely difficult for us to carry out related experiments. Therefore, further validation in multicenter, prospective cohort studies with new experimental validation is needed to support the conclusions of this research. Second, the definition of GBM has been change based on the 2021 WHO Classification of Tumors of the Central Nervous System. The common diffuse gliomas of adults were divided into only 3 types: Astrocytoma, IDH-mutant; Oligodendroglioma, IDH-mutant and 1p/19q-codeleted; and Glioblastoma, IDH-wildtype [98]. The classification of glioblastoma, IDH wild type includes the category of glioblastoma, IDH wild type defined in the 2016 World Health Organization Classification of Tumors of the Central Nervous System. In addition, diffuse astrocytoma, if accompanied by microvascular proliferation or necrosis or TERT promoter mutation or EGFR gene amplification or + 7/-10 chromosome copy number changes, will also be included in glioblastoma, IDH wild type category. Our research data of GBM patients were derived from TCGA database and the Chinese Glioma Genome Atlas (CGGA) database in which patients were classified according to the 2007/2016 WHO classification system [99, 100]. It is really hard to reclassify patients obtained from the previous data with the new WHO classification, due to the complicated new diagnostic criteria with various mutation information and potential diagnostic bias in the second classification. Therefore, analysis of CDI in GBM patients classified by the latest WHO Classification is warranted subsequently with new constructed cohort. Third, an immunotherapy cohort is needed to validate the relationship between CDI and immunotherapy response for the limited number of patients under immunotherapy.

In conclusion, this research provides a potential approach for screening patients at higher risks of mortality based on the cell-death-based gene signature. Moreover, CDI was significantly associated with immune cell infiltration as well as ICB treatment key genes in GBM. Thus, this study brings a novel signature to promote the individualized prediction of overall survival and deepen the understanding of the TME in GBM, further providing promising clinical applications in GBM therapy.

## Supplementary Information

**Additional file1: Figure S1. **Explorations of candidate drugs, which might be capable of targeting the A) high CDI patients and B) low CDI patients by Connectivity Map analysis.

**Additional file2: Table S1. **A list of Apoptosis genes analyzed in this study.

**Additional file3: Table S2. **A list of Necrosis genes analyzed in this study.

**Additional file4: Table S3. **A list of Autophagy genes analyzed in this study.

**Additional file5: Table S4. **Upregulated genes (621 genes) expressed at >1‐fold in high risk group.

**Additional file6: Table S5. **Upregulated genes (148 genes) expressed at >1‐fold in low risk group.

**Additional file7: Table S6. **Univariate and multivariate cox proportional hazards analysis of clinicopathological variables based on PFS in the TCGA GBM training cohort. **Table S7**. Potentialtherapeutic drugs with a score less than -80 for high CDI patients predicted byCmap. **Table S8.** Potentialtherapeutic drugs with a score higher than 80 for low CDI patients predicted byCmap 

## Data Availability

The data supporting this study's findings are openly available in the TCGA dataset, which can be retrieved from GlioVis (http://gliovis.bioin fo.cnio.es/) , and the CGGA database (http://www.cgga.org.cn).

## References

[1] Louis DN, Perry A, Reifenberger G, von Deimling A, Figarella-Branger D, Cavenee WK (2016). The 2016 World Health Organization Classification of Tumors of the Central Nervous System: a summary. Acta Neuropathol.

[2] Wen PY, Kesari S (2008). Malignant gliomas in adults. N Engl J Med.

[3] Nguyen HM, Guz-Montgomery K, Lowe DB, Saha D (2021). Pathogenetic Features and Current Management of Glioblastoma. Cancers (Basel).

[4] Delgado-Martín B, Medina M (2020). Advances in the Knowledge of the Molecular Biology of Glioblastoma and Its Impact in Patient Diagnosis, Stratification, and Treatment. Adv Sci (Weinh).

[5] Witthayanuwat S, Pesee M, Supaadirek C, Supakalin N, Thamronganantasakul K, Krusun S (2018). Survival Analysis of Glioblastoma Multiforme. Asian Pac J Cancer Prev.

[6] Jain KK (2018). A Critical Overview of Targeted Therapies for Glioblastoma. Front Oncol.

[7] Touat M, Idbaih A, Sanson M, Ligon KL (2017). Glioblastoma targeted therapy: updated approaches from recent biological insights. Ann Oncol.

[8] Huang B, Zhang H, Gu L, Ye B, Jian Z, Stary C (2017). Advances in Immunotherapy for Glioblastoma Multiforme. J Immunol Res.

[9] Shergalis A, Bankhead A, Luesakul U, Muangsin N, Neamati N (2018). Current Challenges and Opportunities in Treating Glioblastoma. Pharmacol Rev.

[10] Yu MW, Quail DF (2021). Immunotherapy for Glioblastoma: Current Progress and Challenge. Front Immunol.

[11] Galluzzi L, Vitale I, Warren S, Adjemian S, Agostinis P, Martinez AB (2020). Consensus guidelines for the definition, detection and interpretation of immunogenic cell death. J Immunother Cancer.

[12] Giraldo NA, Sanchez-Salas R, Peske JD, Vano Y, Becht E, Petitprez F (2019). The clinical role of the TME in solid cancer. Br J Cancer.

[13] Galluzzi L, Garg AD (2021). Immunology of Cell Death in Cancer Immunotherapy. Cells.

[14] Galluzzi L, Vitale I, Aaronson SA, Abrams JM, Adam D, Agostinis P (2018). Molecular mechanisms of cell death: recommendations of the Nomenclature Committee on Cell Death 2018. Cell Death Differ.

[15] Nagata S, Tanaka M (2017). Programmed cell death and the immune system. Nat Rev Immunol.

[16] He C, Klionsky DJ (2009). Regulation mechanisms and signaling pathways of autophagy. Annu Rev Genet.

[17] Mariño G, Niso-Santano M, Baehrecke EH, Kroemer G (2014). Self-consumption: the interplay of autophagy and apoptosis. Nat Rev Mol Cell Biol.

[18] Elmore S (2007). Apoptosis: a review of programmed cell death. Toxicol Pathol.

[19] Fleisher TA (1997). Apoptosis. Ann Allergy Asthma Immunol.

[20] Green DR, Llambi F (2015). Cell Death Signaling. Cold Spring Harb Perspect Biol.

[21] Friedmann-Morvinski D (2014). Glioblastoma heterogeneity and cancer cell plasticity. Crit Rev Oncog.

[22] Wang Q, Hu B, Hu X, Kim H, Squatrito M, Scarpace L (2017). Tumor Evolution of Glioma-Intrinsic Gene Expression Subtypes Associates with Immunological Changes in the Microenvironment. Cancer Cell.

[23] Herbst RS, Morgensztern D, Boshoff C (2018). The biology and management of non-small cell lung cancer. Nature.

[24] Yang T, Kong Z, Ma W (2021). PD-1/PD-L1 immune checkpoint inhibitors in glioblastoma: clinical studies, challenges and potential. Hum Vaccin Immunother.

[25] McGranahan T, Therkelsen KE, Ahmad S, Nagpal S (2019). Current State of Immunotherapy for Treatment of Glioblastoma. Curr Treat Options Oncol.

[26] Dapash M, Castro B, Hou D, Lee-Chang C (2021). Current Immunotherapeutic Strategies for the Treatment of Glioblastoma. Cancers (Basel).

[27] Wang Z, Wang Y, Yang T, Xing H, Wang Y, Gao L (2021). Machine learning revealed stemness features and a novel stemness-based classification with appealing implications in discriminating the prognosis, immunotherapy and temozolomide responses of 906 glioblastoma patients. Brief Bioinform.

[28] Li ZH, Guan YL, Zhang GB (2021). Genomic Analysis of Glioblastoma Multiforme Reveals a Key Transcription Factor Signature Relevant to Prognosis and the Immune Processes. Front Oncol.

[29] Silantyev AS, Falzone L, Libra M, Gurina OI, Kardashova KS, Nikolouzakis TK (2019). Current and Future Trends on Diagnosis and Prognosis of Glioblastoma: From Molecular Biology to Proteomics. Cells.

[30] Bowman RL, Wang Q, Carro A, Verhaak RG, Squatrito M (2017). GlioVis data portal for visualization and analysis of brain tumor expression datasets. Neuro Oncol.

[31] Zhao Z, Meng F, Wang W, Wang Z, Zhang C, Jiang T (2017). Comprehensive RNA-seq transcriptomic profiling in the malignant progression of gliomas. Sci Data.

[32] Zhao S, Fung-Leung WP, Bittner A, Ngo K, Liu X (2014). Comparison of RNA-Seq and microarray in transcriptome profiling of activated T cells. PLoS One.

[33] Zhang H, Meltzer P, Davis S (2013). RCircos: an R package for Circos 2D track plots. BMC Bioinformatics.

[34] Mayakonda A, Lin DC, Assenov Y, Plass C, Koeffler HP (2018). Maftools: efficient and comprehensive analysis of somatic variants in cancer. Genome Res.

[35] Kanehisa M, Furumichi M, Sato Y, Ishiguro-Watanabe M, Tanabe M (2021). KEGG: integrating viruses and cellular organisms. Nucleic Acids Res.

[36] Smyth GK, Michaud J, Scott HS (2005). Use of within-array replicate spots for assessing differential expression in microarray experiments. Bioinformatics.

[37] Liberzon A, Subramanian A, Pinchback R, Thorvaldsdóttir H, Tamayo P, Mesirov JP (2011). Molecular signatures database (MSigDB) 3.0. Bioinformatics.

[38] Szklarczyk D, Gable AL, Lyon D, Junge A, Wyder S, Huerta-Cepas J (2019). STRING v11: protein-protein association networks with increased coverage, supporting functional discovery in genome-wide experimental datasets. Nucleic Acids Res.

[39] Li T, Fu J, Zeng Z, Cohen D, Li J, Chen Q (2020). TIMER2.0 for analysis of tumor-infiltrating immune cells. Nucleic Acids Res.

[40] Yi M, Nissley DV, McCormick F, Stephens RM (2020). ssGSEA score-based Ras dependency indexes derived from gene expression data reveal potential Ras addiction mechanisms with possible clinical implications. Sci Rep.

[41] Newman AM, Liu CL, Green MR, Gentles AJ, Feng W, Xu Y (2015). Robust enumeration of cell subsets from tissue expression profiles. Nat Methods.

[42] Aran D, Hu Z, Butte AJ (2017). xCell: digitally portraying the tissue cellular heterogeneity landscape. Genome Biol.

[43] Shi J, Jiang D, Yang S, Zhang X, Wang J, Liu Y (2020). LPAR1, Correlated With Immune Infiltrates, Is a Potential Prognostic Biomarker in Prostate Cancer. Front Oncol.

[44] Finotello F, Mayer C, Plattner C, Laschober G, Rieder D, Hackl H (2019). Molecular and pharmacological modulators of the tumor immune contexture revealed by deconvolution of RNA-seq data. Genome Med.

[45] Li T, Fan J, Wang B, Traugh N, Chen Q, Liu JS (2017). TIMER: A Web Server for Comprehensive Analysis of Tumor-Infiltrating Immune Cells. Cancer Res.

[46] Saerens M, Brusselaers N, Rottey S, Decruyenaere A, Creytens D, Lapeire L (2021). Immune checkpoint inhibitors in treatment of soft-tissue sarcoma: A systematic review and meta-analysis. Eur J Cancer.

[47] Bai Y, Lin H, Chen J, Wu Y, Yu S (2021). Identification of Prognostic Glycolysis-Related lncRNA Signature in Tumor Immune Microenvironment of Hepatocellular Carcinoma. Front Mol Biosci.

[48] Raudvere U, Kolberg L, Kuzmin I, Arak T, Adler P, Peterson H (2019). g:Profiler: a web server for functional enrichment analysis and conversions of gene lists (2019 update). Nucleic Acids Res.

[49] Szklarczyk D, Gable AL, Nastou KC, Lyon D, Kirsch R, Pyysalo S (2021). The STRING database in 2021: customizable protein-protein networks, and functional characterization of user-uploaded gene/measurement sets. Nucleic Acids Res.

[50] Chin CH, Chen SH, Wu HH, Ho CW, Ko MT, Lin CY (2014). cytoHubba: identifying hub objects and sub-networks from complex interactome. BMC Syst Biol.

[51] Hänzelmann S, Castelo R, Guinney J (2013). GSVA: gene set variation analysis for microarray and RNA-seq data. BMC Bioinformatics.

[52] Kanehisa M (2019). Toward understanding the origin and evolution of cellular organisms. Protein Sci.

[53] Kanehisa M, Goto S (2000). KEGG: kyoto encyclopedia of genes and genomes. Nucleic Acids Res.

[54] Subramanian A, Narayan R, Corsello SM, Peck DD, Natoli TE, Lu X (2017). A Next Generation Connectivity Map: L1000 Platform and the First 1,000,000 Profiles. Cell.

[55] Lin W, Wu S, Chen X, Ye Y, Weng Y, Pan Y (2020). Characterization of Hypoxia Signature to Evaluate the Tumor Immune Microenvironment and Predict Prognosis in Glioma Groups. Front Oncol.

[56] Nakano I (2015). Stem cell signature in glioblastoma: therapeutic development for a moving target. J Neurosurg.

[57] Deng S, Zheng Y, Mo Y, Xu X, Li Y, Zhang Y (2021). Ferroptosis Suppressive Genes Correlate with Immunosuppression in Glioblastoma. World Neurosurg.

[58] D'Orsi B, Niewidok N, Düssmann H, Prehn JHM (2021). Mitochondrial Carrier Homolog 2 Functionally Co-operates With BH3 Interacting-Domain Death Agonist in Promoting Ca(2+)-Induced Neuronal Injury. Front Cell Dev Biol.

[59] Zhang J, Singh N, Robinson-Taylor KS, Dorsett-Martin WA, Morris MW, Earl TM (2015). Hepatocyte autophagy is linked to C/EBP-homologous protein, Bcl2-interacting mediator of cell death, and BH3-interacting domain death agonist gene expression. J Surg Res.

[60] Park HJ, Kim JW, Cho BS, Chung JH (2014). Association of BH3 interacting domain death agonist (BID) gene polymorphisms with proteinuria of immunoglobulin A nephropathy. Scand J Clin Lab Invest.

[61] Wang N, Zhu P, Huang R, Sun L, Dong D, Gao Y (2021). Suppressing TRAP1 sensitizes glioblastoma multiforme cells to temozolomide. Exp Ther Med.

[62] Anand N, Murthy S, Amann G, Wernick M, Porter LA, Cukier IH (2002). Protein elongation factor EEF1A2 is a putative oncogene in ovarian cancer. Nat Genet.

[63] Yang S, Lu M, Chen Y, Meng D, Sun R, Yun D (2015). Overexpression of eukaryotic elongation factor 1 alpha-2 is associated with poorer prognosis in patients with gastric cancer. J Cancer Res Clin Oncol.

[64] Kawamura M, Endo C, Sakurada A, Hoshi F, Notsuda H, Kondo T (2014). The prognostic significance of eukaryotic elongation factor 1 alpha-2 in non-small cell lung cancer. Anticancer Res.

[65] Pinke DE, Kalloger SE, Francetic T, Huntsman DG, Lee JM (2008). The prognostic significance of elongation factor eEF1A2 in ovarian cancer. Gynecol Oncol.

[66] Giudici F, Petracci E, Nanni O, Bottin C, Pinamonti M, Zanconati F (2019). Elevated levels of eEF1A2 protein expression in triple negative breast cancer relate with poor prognosis. PLoS One.

[67] Comoglio PM, Trusolino L, Boccaccio C (2018). Known and novel roles of the MET oncogene in cancer: a coherent approach to targeted therapy. Nat Rev Cancer.

[68] Gdynia G, Grund K, Eckert A, Böck BC, Funke B, Macher-Goeppinger S (2007). Basal caspase activity promotes migration and invasiveness in glioblastoma cells. Mol Cancer Res.

[69] Sohn EJ, Shin MJ, Eum WS, Kim DW, Yong JI, Ryu EJ (2016). Tat-NOL3 protects against hippocampal neuronal cell death induced by oxidative stress through the regulation of apoptotic pathways. Int J Mol Med.

[70] Farshchian M, Kivisaari A, Ala-Aho R, Riihilä P, Kallajoki M, Grénman R (2011). Serpin peptidase inhibitor clade A member 1 (SerpinA1) is a novel biomarker for progression of cutaneous squamous cell carcinoma. Am J Pathol.

[71] Jun S, Lee S, Kim HC, Ng C, Schneider AM, Ji H (2013). PAF-mediated MAPK signaling hyperactivation via LAMTOR3 induces pancreatic tumorigenesis. Cell Rep.

[72] Wang Y, Zhao W, Xiao Z, Guan G, Liu X, Zhuang M (2020). A risk signature with four autophagy-related genes for predicting survival of glioblastoma multiforme. J Cell Mol Med.

[73] Wang QW, Liu HJ, Zhao Z, Zhang Y, Wang Z, Jiang T (2020). Prognostic Correlation of Autophagy-Related Gene Expression-Based Risk Signature in Patients with Glioblastoma. Onco Targets Ther.

[74] Yoshimura S, Sano E, Hanashima Y, Yamamuro S, Sumi K, Ueda T (2019). IFN-β sensitizes TRAIL-induced apoptosis by upregulation of death receptor 5 in malignant glioma cells. Oncol Rep.

[75] Pombo Antunes AR, Scheyltjens I, Duerinck J, Neyns B, Movahedi K, Van Ginderachter JA: Understanding the glioblastoma immune microenvironment as basis for the development of new immunotherapeutic strategies. Elife 2020, 9. 10.7554/eLife.52176 PMC7000215 32014107

[76] De Luca C, Colangelo AM, Alberghina L, Papa M (2018). Neuro-Immune Hemostasis: Homeostasis and Diseases in the Central Nervous System. Front Cell Neurosci.

[77] Yin J, Valin KL, Dixon ML, Leavenworth JW (2017). The Role of Microglia and Macrophages in CNS Homeostasis, Autoimmunity, and Cancer. J Immunol Res.

[78] Abadi B, Yazdanpanah N, Nokhodchi A, Rezaei N (2021). Smart Biomaterials to Enhance the Efficiency of Immunotherapy in Glioblastoma: State of the Art and Future Perspectives. Adv Drug Deliv Rev.

[79] Gabrusiewicz K, Rodriguez B, Wei J, Hashimoto Y, Healy LM, Maiti SN (2016). Glioblastoma-infiltrated innate immune cells resemble M0 macrophage phenotype. JCI Insight.

[80] Ott M, omaszowski KH, Marisetty A, Kong LY, Wei J, Duna M (2020). Profiling of patients with glioma reveals the dominant immunosuppressive axis is refractory to immune function restoration. JCI Insight.

[81] Chen Z, Hambardzumyan D (2018). Immune Microenvironment in Glioblastoma Subtypes. Front Immunol.

[82] Kong LY, Wu AS, Doucette T, Wei J, Priebe W, Fuller GN (2010). Intratumoral mediated immunosuppression is prognostic in genetically engineered murine models of glioma and correlates to immunotherapeutic responses. Clin Cancer Res.

[83] Hussain SF, Yang D, Suki D, Aldape K, Grimm E, Heimberger AB (2006). The role of human glioma-infiltrating microglia/macrophages in mediating antitumor immune responses. Neuro Oncol.

[84] Fecci PE, Mitchell DA, Whitesides JF, Xie W, Friedman AH, Archer GE (2006). Increased regulatory T-cell fraction amidst a diminished CD4 compartment explains cellular immune defects in patients with malignant glioma. Cancer Res.

[85] Lohr J, Ratliff T, Huppertz A, Ge Y, Dictus C, Ahmadi R (2011). Effector T-cell infiltration positively impacts survival of glioblastoma patients and is impaired by tumor-derived TGF-β. Clin Cancer Res.

[86] Abril-Rodriguez G, Ribas A (2017). SnapShot: Immune Checkpoint Inhibitors. Cancer Cell.

[87] Li B, Chan HL, Chen P (2019). Immune Checkpoint Inhibitors: Basics and Challenges. Curr Med Chem.

[88] Zhao J, Chen AX, Gartrell RD, Silverman AM, Aparicio L, Chu T (2019). Immune and genomic correlates of response to anti-PD-1 immunotherapy in glioblastoma. Nat Med.

[89] Romani M, Pistillo MP, Carosio R, Morabito A, Banelli B (2018). Immune Checkpoints and Innovative Therapies in Glioblastoma. Front Oncol.

[90] Prendergast GC, Smith C, Thomas S, Mandik-Nayak L, Laury-Kleintop L, Metz R (2014). Indoleamine 2,3-dioxygenase pathways of pathogenic inflammation and immune escape in cancer. Cancer Immunol Immunother.

[91] Yu CP, Fu SF, Chen X, Ye J, Ye Y, Kong LD (2018). The Clinicopathological and Prognostic Significance of IDO1 Expression in Human Solid Tumors: Evidence from a Systematic Review and Meta-Analysis. Cell Physiol Biochem.

[92] Touat M, Li YY, Boynton AN, Spurr LF, Iorgulescu JB, Bohrson CL (2020). Mechanisms and therapeutic implications of hypermutation in gliomas. Nature.

[93] Bouffet E, Larouche V, Campbell BB, Merico D, de Borja R, Aronson M (2016). Immune Checkpoint Inhibition for Hypermutant Glioblastoma Multiforme Resulting From Germline Biallelic Mismatch Repair Deficiency. J Clin Oncol.

[94] Delen E, Doğanlar O (2020). The Dose Dependent Effects of Ruxolitinib on the Invasion and Tumorigenesis in Gliomas Cells via Inhibition of Interferon Gamma-Depended JAK/STAT Signaling Pathway. J Korean Neurosurg Soc.

[95] Ding Z, Kloss JM, Tuncali S, Tran NL, Loftus JC (2020). TROY signals through JAK1-STAT3 to promote glioblastoma cell migration and resistance. Neoplasia.

[96] Dao KT, Gotlib J, Deininger MMN, Oh ST, Cortes JE, Collins RH (2020). Efficacy of Ruxolitinib in Patients With Chronic Neutrophilic Leukemia and Atypical Chronic Myeloid Leukemia. J Clin Oncol.

[97] Geribaldi-Doldán N, Hervás-Corpión I, Gómez-Oliva R, Domínguez-García S, Ruiz FA, Iglesias-Lozano I (2021). Targeting Protein Kinase C in Glioblastoma Treatment. Biomedicines.

[98] Louis DN, Perry A, Wesseling P, Brat DJ, Cree IA, Figarella-Branger D (2021). The 2021 WHO Classification of Tumors of the Central Nervous System: a summary. Neuro Oncol.

[99] Zhao Z, Zhang KN, Wang Q, Li G, Zeng F, Zhang Y (2021). Chinese Glioma Genome Atlas (CGGA): A Comprehensive Resource with Functional Genomic Data from Chinese Glioma Patients. Genomics Proteomics Bioinformatics.

[100] Verhaak RG, Hoadley KA, Purdom E, Wang V, Qi Y, Wilkerson MD (2010). Integrated genomic analysis identifies clinically relevant subtypes of glioblastoma characterized by abnormalities in PDGFRA, IDH1, EGFR, and NF1. Cancer Cell.

