# Supplementary material for: Prognostic value and immune relevancy of a combined autophagy-, apoptosis- and necrosis-related gene signature in glioblastoma
Source: BMC Cancer. 2022 Mar 3;22:233. doi: 10.1186/s12885-022-09328-3 (PMC8892733; doi:10.1186/s12885-022-09328-3)

**Supplementary Figure:**

Figure S1. Explorations of candidate drugs, which might be capable of targeting the A) high CDI patients and B) low CDI patients by Connectivity Map analysis.


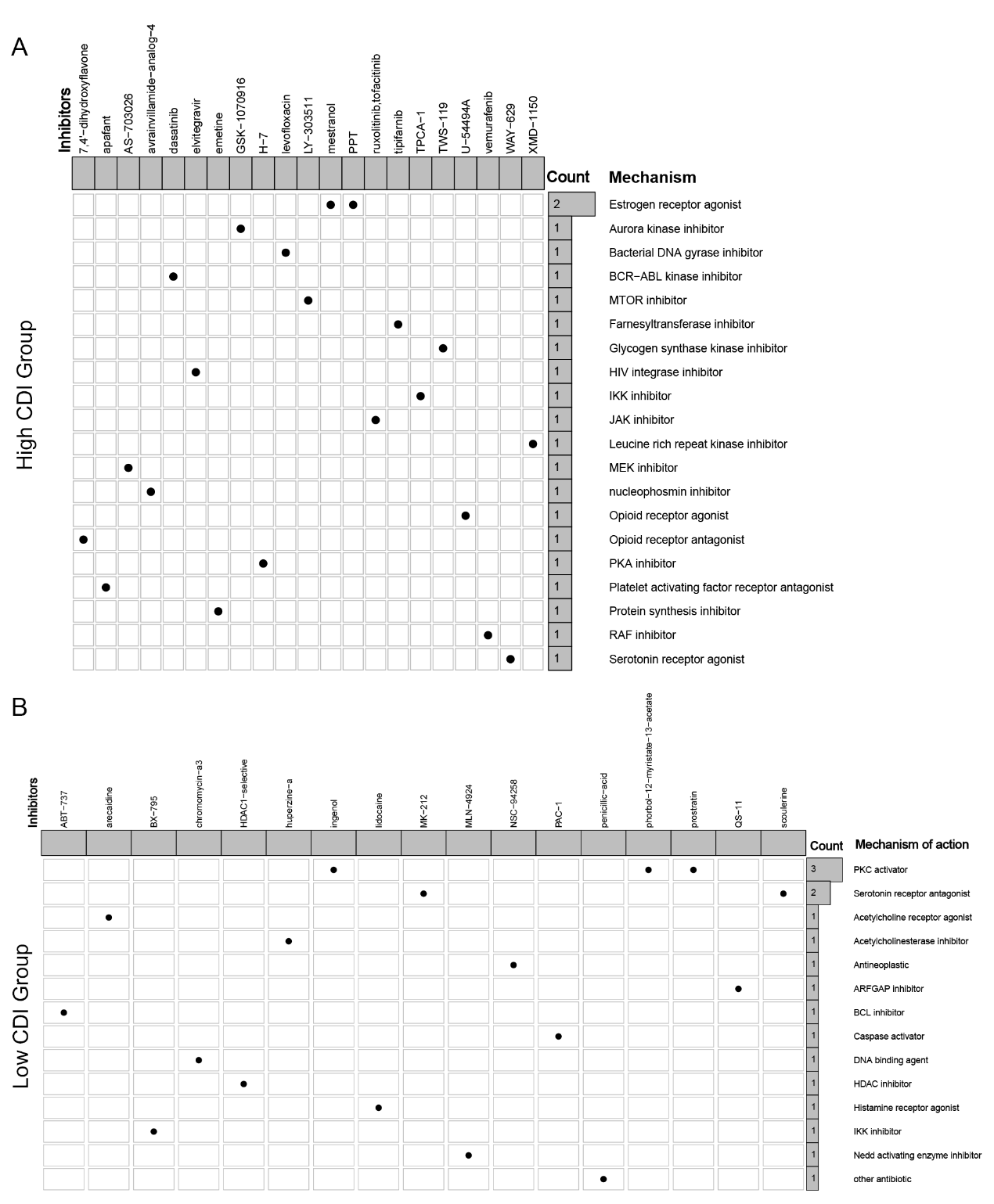

Supplement: Supplementary file 1 — Additional file1: Figure S1. Explorations of candidate drugs, which might be capable of targeting the A) high CDI patients and B) low CDI patients by Connectivity Map analysis. [file 12885_2022_9328_MOESM1_ESM.docx]
