# Supplementary material for: Prognostic value and immune relevancy of a combined autophagy-, apoptosis- and necrosis-related gene signature in glioblastoma
Source: BMC Cancer. 2022 Mar 3;22:233. doi: 10.1186/s12885-022-09328-3 (PMC8892733; doi:10.1186/s12885-022-09328-3)
Supplement: Supplementary file 7 — Additional file7: Table S6. Univariate and multivariate cox proportional hazards analysis of clinicopathological variables based on PFS in the TCGA GBM training cohort. Table S7. Potentialtherapeutic drugs with a score less than -80 for high CDI patients predicted byCmap. Table S8. Potentialtherapeutic drugs with a score higher than 80 for low CDI patients predicted byCmap [file 12885_2022_9328_MOESM7_ESM.docx]

**Supplementary tables:**

| Table S6. Univariate andmultivariate cox proportional hazards analysis of clinicopathological variables based on PFS in the TCGA GBM training cohort | | | | | | | |
| --- | --- | --- | --- | --- | --- | --- | --- |
| Variables | TCGA training cohort（n=518) | | | | | | |
|  | Univariate analysis | | |  | Multivariate analysis | | |
|  | HR | 95%CI | p.value |  | HR | 95%CI | p.value |
| Age | 1.0185 | 1.0116-1.0255 | 1.30E-07 |  | **1.0255** | **1.0142-1.037** | **9.16E-06** |
| Gender | 1.1812 | 0.97414-1.4323 | 0.090353 |  |  |  |  |
| surgery | 1.2217 | 0.92057-1.6213 | 0.16551 |  |  |  |  |
| KPS | 0.80065 | 0.61594-1.0408 | 0.096638 |  |  |  |  |
| TMZ chemotherapy | 0.85703 | 0.70303-1.0448 | 0.12688 |  |  |  |  |
| Radiotherapy | 0.57899 | 0.44414-0.75477 | 5.35E-05 |  | **0.41953** | **0.29863-0.58937** | **5.48E-07** |
| Standard chemoradiation | 0.94799 | 0.77959-1.1528 | 0.5925 |  |  |  |  |
| G-CIMP status | 0.32815 | 0.22444-0.47976 | 8.93E-09 |  | 0.24843 | 0.032817-1.8806 | 0.17753 |
| TCGA Subtype | 0.89824 | 0.82805-0.97438 | 0.009733 |  | 1.1324 | 0.98834-1.2975 | 0.073298 |
| IDH mutation status | 0.33326 | 0.20655-0.5377 | 6.73E-06 |  | 2.6072 | 0.33515-20.281 | 0.35992 |
| MGMT promoter methylation status | 0.72604 | 0.57131-0.92268 | 0.008843 |  | 0.77213 | 0.5897-1.011 | 0.060049 |
| CDI high risk | 2.1767 | 1.7857-2.6532 | 1.36E-14 |  | **2.0988** | **1.5411-2.8583** | **2.54E-06** |
|  |  |  |  |  |  |  |  |

| Table S7. Potential therapeutic drugs with a score less than -80 for high CDI patients predicted by Cmap | | | |
| --- | --- | --- | --- |
| **Drug Name** | **Mechanism of Action** | **Target** | **Score** |
| Tipifarnib | Farnesyltransferase inhibitor | FNTA, FNTB | -96.1 |
| Tofacitinib | JAK inhibitor | JAK3, JAK1, JAK2, CYP2C19, TYK2 | -95.81 |
| GSK-1070916 | Aurora kinase inhibitor | AURKB, AURKC, AURKA, CYP2D6, CYP3A4 | -94.93 |
| Mestranol | Estrogen receptor agonist | ESR1 | -94.48 |
| Ruxolitinib | JAK inhibitor | JAK1, JAK2, TYK2, JAK3 | -93.19 |
| XMD-1150 | Leucine rich repeat kinase inhibitor | LRRK2 | -92.64 |
| TPCA-1 | IKK inhibitor | IKBKB | -91.9 |
| PPT | Estrogen receptor agonist | ESR1 | -91.84 |
| 7,4'-dihydroxyflavone | Opioid receptor antagonist | CYP19A1 | -91.29 |
| Avrainvillamide-analog-4 | Nucleophosmin inhibitor | NPM1 | -89.8 |
| Vemurafenib | RAF inhibitor | BRAF, CYP2C19, CYP3A4, CYP3A5, RAF1 | -89.23 |
| Emetine | Protein synthesis inhibitor | RPS2 | -87.62 |
| LY-303511 | Casein kinase inhibitor, MTOR inhibitor, PI3K inhibitor | CSNK2A1, CSNK2A2, CSNK2B, MTOR | -86.6 |
| H-7 | PKA inhibitor | PKIA, PRKACA, PRKAR1A | -86.17 |
| U-54494A | Opioid receptor agonist |  | -86.15 |
| AS-703026 | MEK inhibitor | MAP2K1, MAP2K2 | -85.83 |
| Elvitegravir | HIV integrase inhibitor |  | -85.73 |
| Levofloxacin | Bacterial DNA gyrase inhibitor | TOP2A | -83.15 |
| Dasatinib | BCR-ABL kinase inhibitor, Ephrin inhibitor, KIT inhibitor, PDGFR receptor inhibitor, SRC inhibitor, Tyrosine kinase inhibitor | ABL1, FYN, LCK, SRC, KIT, YES1, BCR, EPHA2, LYN, PDGFRB, ABL2, BTK, DDR1, DDR2, PDGFRA, STAT5B | -82.02 |
| TWS-119 | Glycogen synthase kinase inhibitor | GSK3B, JUN, MYC | -81.86 |
| Apafant | Platelet activating factor receptor antagonist | PTAFR | -81.84 |
| WAY-629 | Serotonin receptor agonist | HTR2C | -81.79 |
|  |  |  |  |

| Table S8. Potential therapeutic drugs with a score higher than 80 for low CDI patients predicted by Cmap | | |  |
| --- | --- | --- | --- |
| **Drug Name** | **Mechanism of Action** | **Target** | **Score** |
| Ingenol | PKC activator | PRKCD, PRKCE | 96.05 |
| Prostratin | PKC activator | PRKCA, PRKCB, PRKCD, PRKCE, PRKCG, PRKCH, PRKCQ | 95.81 |
| Scoulerine | Adrenergic receptor antagonist, GABA receptor antagonist, Serotonin receptor antagonist | ADRA1D, ADRA2A, GABRA1 | 95.29 |
| NSC-94258 | Antineoplastic | AKR1B1, CYP19A1, HSD17B1 | 93.08 |
| Arecaidine | Acetylcholine receptor agonist | CHRM1, CHRM2, CHRM3, CHRM4 | 92.67 |
| BX-795 | IKK inhibitor | PDPK1, CDK2, CHEK1, GSK3B, IKBKE, KDR, PDK1, TBK1 | 92.53 |
| Phorbol-12-myristate-13-acetate | PKC activator | CD4, KCNT2, PRKCA, TRPV4 | 90.49 |
| PAC-1 | Caspase activator | CASP3 | 89.86 |
| QS-11 | ARFGAP inhibitor | ARFGAP1 | 89.55 |
| Huperzine-a | Acetylcholinesterase inhibitor | ACHE | 87.72 |
| ABT-737 | BCL inhibitor | BCL2, BCL2L1, BCL2L2 | 87.35 |
| Chromomycin-a3 | DNA binding agent |  | 84.94 |
| MLN-4924 | Nedd activating enzyme inhibitor | NAE1, UBA3 | 84.71 |
| HDAC1-selective | HDAC inhibitor |  | 83.74 |
| SA-63133 | NA | CSNK1E, CSNK1A1, CSNK1D, CSNK1G2 | 83.3 |
| MK-212 | Serotonin receptor agonist | HTR2A, HTR2B, HTR2C | 80.99 |
| Lidocaine | Histamine receptor agonist | SCN5A, EGFR, SCN10A, SCN9A | 80.93 |
| Penicillic-acid | other antibiotic |  | 80.48 |
|  |  |  |  |
